# A muscle-liver-fat signalling axis is essential for central control of adaptive adipose remodelling

**DOI:** 10.1038/ncomms7693

**Published:** 2015-04-01

**Authors:** Noriaki Shimizu, Takako Maruyama, Noritada Yoshikawa, Ryo Matsumiya, Yanxia Ma, Naoki Ito, Yuki Tasaka, Akiko Kuribara-Souta, Keishi Miyata, Yuichi Oike, Stefan Berger, Günther Schütz, Shin’ichi Takeda, Hirotoshi Tanaka

**Affiliations:** 1 Department of Rheumatology and Allergy, IMSUT Hospital, The Institute of Medical Science, The University of Tokyo, 4-6-1, Shirokanedai, Minato-ku, Tokyo 108-8639, Japan; 2 Department of Molecular Therapy, National Institute of Neuroscience, National Center of Neurology and Psychiatry, 4-1-1, Ogawa-higashi, Kodaira, Tokyo 187-8502, Japan; 3 Department of Molecular Genetics, Graduate School of Medical Sciences, Kumamoto University, 1-1-1, Honjo, Chuo-ku, Kumamoto 860-8556, Japan; 4 Division of Molecular Biology of the Cell I, German Cancer Research Center, Im Neuenheimer Feld 280, Heidelberg 69120, Germany; 5 Division of Rheumatology, Center for Antibody and Vaccine, IMSUT Hospital, The Institute of Medical Science, The University of Tokyo, 4-6-1, Shirokanedai, Minato-ku, Tokyo 108-8639, Japan

## Abstract

Skeletal muscle has a pleiotropic role in organismal energy metabolism, for example, by storing protein as an energy source, or by excreting endocrine hormones. Muscle proteolysis is tightly controlled by the hypothalamus-pituitary-adrenal signalling axis via a glucocorticoid-driven transcriptional programme. Here we unravel the physiological significance of this catabolic process using skeletal muscle-specific glucocorticoid receptor (*GR*) knockout (GRmKO) mice. These mice have increased muscle mass but smaller adipose tissues. Metabolically, GRmKO mice show a drastic shift of energy utilization and storage in muscle, liver and adipose tissues. We demonstrate that the resulting depletion of plasma alanine serves as a cue to increase plasma levels of fibroblast growth factor 21 (FGF21) and activates liver-fat communication, leading to the activation of lipolytic genes in adipose tissues. We propose that this skeletal muscle-liver-fat signalling axis may serve as a target for the development of therapies against various metabolic diseases, including obesity.

## Abstract (web summary)

Skeletal muscle proteolysis can affect organismal energy homeostasis. Here, the authors provide molecular insight into this process by showing that muscle-derived alanine acts as a signal that triggers FGF21 secretion from the liver, which then regulates lipolysis and browning of white fat tissue.

## Graphical abstract

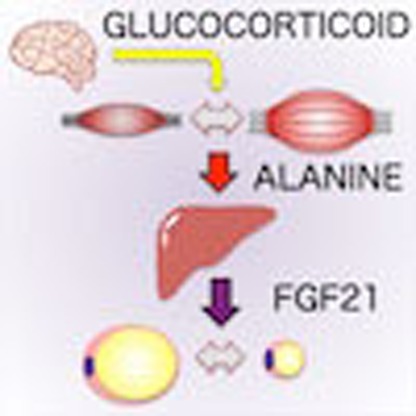

Mammals store various macromolecules, for example, carbohydrates, proteins and lipids, as fuels, and regulate the energy fluxes to meet the metabolic demands. Fuel storage organs, for example, liver, skeletal muscle and adipose tissues, communicating with each other, determine fuel selection and delivery under the strict control of neuroendocrine system^1^,^2^. Dysregulation of this energy control often results in human metabolic disorders including obesity, diabetes and metabolic syndrome. How this control is regulated for adaptive adjustment of systemic metabolism, however, remains unknown.

Skeletal muscle not only functions as a locomotive apparatus but also delivers the protein breakdown products, for example, alanine, to the liver as substrates for gluconeogenesis (glucose-alanine cycle)^3^,^4^,^5^. Muscle proteolysis is an active process tightly controlled by specific signalling pathways and transcriptional programmes, involving ubiquitin-proteasome and autophagy-lysosome machineries^6^,^7^. Glucocorticoid receptor (*GR*) transcriptionally controls expression levels of components in the both machineries as well as those of key transcription factors for catabolism, FoxOs and KLF15 (ref. 8).

Here we show that skeletal muscle-specific *GR* knockout (GRmKO) mice have increased muscle mass but smaller adipose tissues, accompanied with a drastic shift of gene expression in the muscle, liver and adipose tissues. We demonstrate that the resulting depletion of plasma alanine serves as a cue to increase plasma levels of fibroblast growth factor 21 (FGF21) and activates liver-fat communication, leading to the activation of lipolytic genes in adipose tissues. We propose that this skeletal muscle-liver-fat signalling axis controls organismal energy distribution and may serve as a target for the development of therapies against various metabolic diseases, including obesity.

## Results

### *GR* ablation in skeletal muscle causes muscle hypertrophy

**Figure 1:**
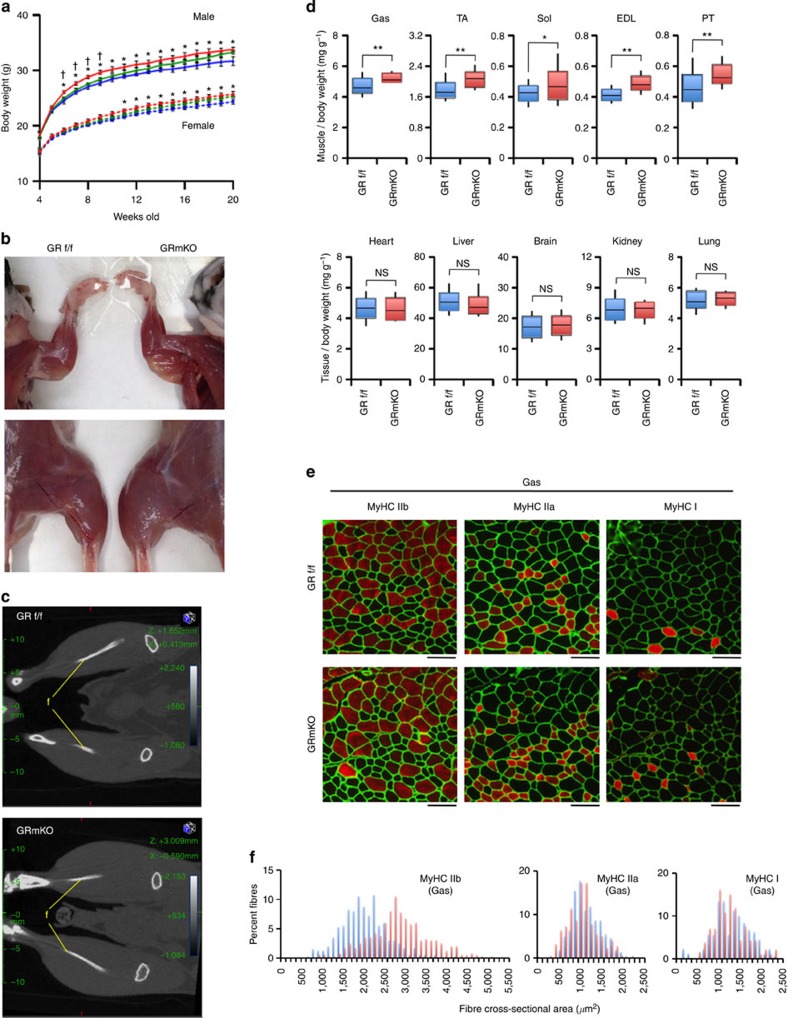
Skeletal muscle hypertrophy caused by *GR* ablation in skeletal muscle. (**a**) Body weight of *ad libitum*-fed male (solid lines) and female (dotted lines) GRf/f (blue), GRmKO (red) and *GR^flox/+^ACTA1-Cre^+^* (hetero GRmKO, green) mice. Error bars represent mean±s.e.m. (*n*=16). *P<0.05 between GRf/f and GRmKO; ^†^*P*<0.05 between GRmKO and hetero GRmKO determined by two-tailed Student’s *t*-test for unpaired data. (**b**) Representative photographs of 18-week-old male GRf/f and GRmKO mice. (**c**) Representative computed tomography (CT) coronal section images of 18-week-old male GRf/f and GRmKO mice. Fibulae are indicated as f. (**d**) Weight of tissues from 20-week-old male GRf/f and GRmKO mice. Data are shown as boxplots of ratio to body weight (*n*=16). Muscles are abbreviated as Gas (gastrocnemius), TA (tibialis anterior), Sol (soleus), EDL (extensor digitorum longus) and PT (plantaris). Whiskers show the minimum and maximum of all the data, boxes show one standard deviation above and below the mean of the data, and lines inside the boxes show the median of the data. *P<0.05; **P<0.01 determined by two-tailed Student’s *t*-test for unpaired data. NS, not significant. (**e**) Representative images of immunostaining for type IIb, IIa and I myosin heavy chain (red in the panels indicated as MyHC IIb, MyHC IIa and MyHC I, respectively) and type IV collagen (green) of serial transverse cryosections of Gas from 20-week-old male GRf/f and GRmKO mice. Scale bars represent 100 μm. (**f**) Myofibre cross-sectional area (CSA) distribution in Gas from 20-week-old male GRf/f (blue) and GRmKO (red) are shown as frequency histograms (300<*n*<330, from three independent animals of each genotype).

Skeletal muscle-specific deletion of *GR* in GRmKO mice was confirmed in a series of experiments (Supplementary Fig. 1a–d). GRmKO mice showed slight decrease in locomotor activity, subtle elevation in body temperature, and significant decrease in O_2_ consumption rate, CO_2_ production rate and energy expenditure, compared with GRf/f mice (Supplementary Fig. 1e–j). After 7-day treatment with dexamethasone (DEX), GR-target gene expression in the liver was upregulated in both mice (Supplementary Fig. 2a). Meanwhile, GR-target genes in skeletal muscle, several of which were related to muscle protein degradation (*KLF15, FoxOs, MuRF1, atrogin-1, Bnip3* and *LC3*)^7^,^8^ were not upregulated in gastrocnemius muscle of GRmKO (Supplementary Fig. 2b). Also, repression of mammalian target of rapamycin activity^7^,^8^ (Supplementary Fig. 2c) and reduced cross-sectional area of myofibres (Supplementary Fig. 2d) were exclusively observed in GRf/f mice. GRmKO mice grew normally but appeared to be heavier than GRf/f both in male and female (Fig. 1a). Gross appearance (Fig. 1b and Supplementary Fig. 3) and computed tomography (CT) analysis (Fig. 1c) revealed that GRmKO mice showed increased skeletal muscle mass. Every skeletal muscle so far examined was heavier in GRmKO while other tissues were not (Fig. 1d). Cross-sectional area of myofibres, especially glucocorticoid-sensitive type IIb fibres^7^,^8^, were larger in GRmKO (Fig. 1e,f and Supplementary Fig. 4).

### Energy supply via GR-mediated muscle protein catabolism

**Figure 2:**
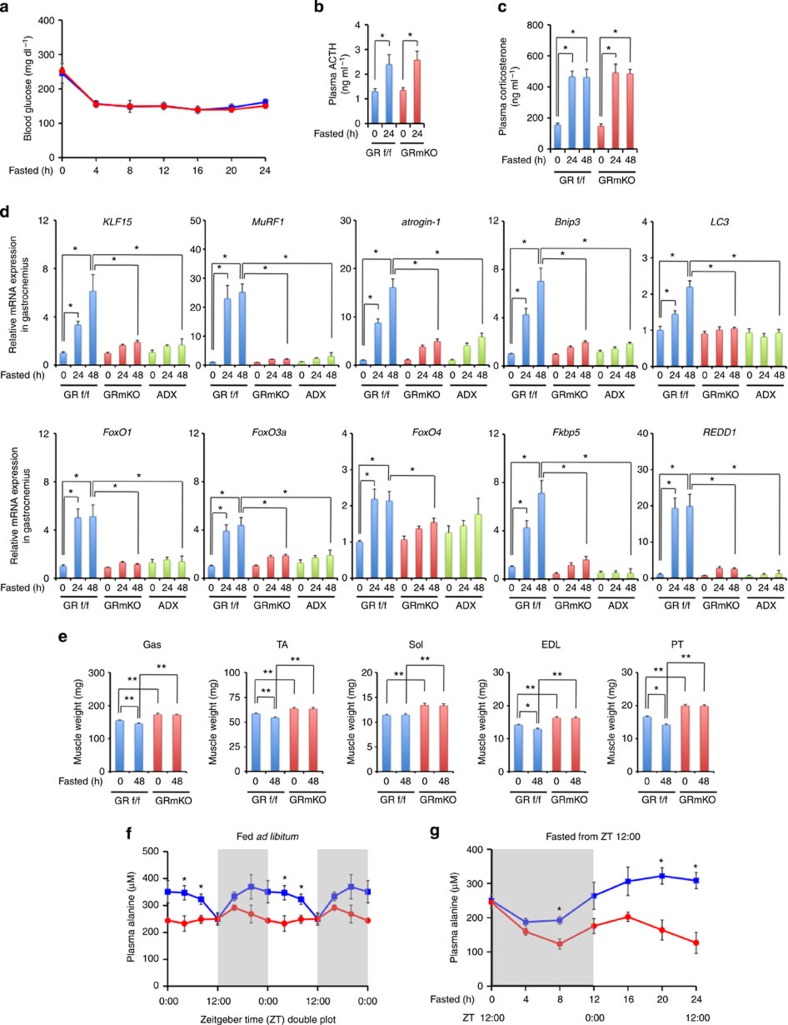
Tuning of plasma alanine concentration via GR-mediated skeletal muscle protein catabolism. (**a**–**c**) Blood glucose (**a**), plasma adrenocorticotropic hormone (ACTH, **b**) and corticosterone (**c**) levels of 20-week-old male GRf/f (blue) and GRmKO (red) mice after fasting for the indicated time periods. Error bars represent mean±s.e.m. (*n*=8). *P<0.05 determined by two-tailed Student’s *t*-test for unpaired data. (**d**) Expression levels of the indicated mRNA in Gas (gastrocnemius) from 20-week-old male GRf/f, GRmKO and adrenalectomized C57BL/6J (adrenalectomy (ADX)) mice after fasting for the indicated time periods. Data are normalized to *36B4* mRNA levels and are shown as fold induction to expression levels in fed (0 h) GRf/f mice. Error bars represent mean±s.e.m. (*n*=8). *P<0.05 determined by two-tailed Student’s *t*-test for unpaired data. (**e**) Weight of skeletal muscles from *ad libitum*-fed and 48 h-fasted 20-week-old male GRf/f and GRmKO mice. Error bars represent mean±s.e.m. (*n*=12). *P<0.05; **P<0.01 determined by two-tailed Student’s *t*-test for unpaired data. (**f**,**g**) Plasma alanine levels of 20-week-old male GRf/f (blue) and GRmKO (red) mice, which were *ad libitum*-fed (**f**) and fasted for the indicated time periods from zeitgeber time (ZT) 12:00 (**g**). Error bars represent mean±s.e.m. (*n*=4). *P<0.05 between GRf/f and GRmKO determined by two-tailed Student’s *t*-test for unpaired data.

**Figure 3:**
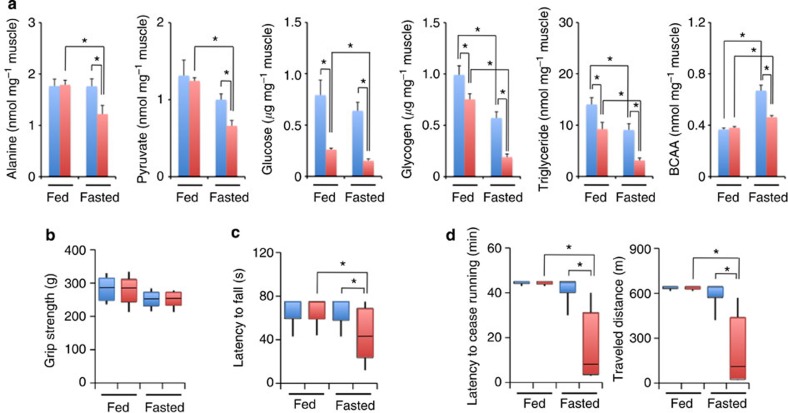
Inefficient energy production in *GR*-ablated skeletal muscle during fasting. (**a**) Intramuscular levels of alanine, pyruvate, glucose, glycogen, triglyceride and branched chain amino acids (BCAA) in Gas (gastrocnemius) from fed or 24 h-fasted 20-week-old male GRf/f (blue) and GRmKO (red) mice. Error bars represent mean±s.e.m. (*n*=6). *P<0.05 determined by two-tailed Student’s *t*-test for unpaired data. (**b**,**c**) Maximum grip strength of limbs (**b**) and endurance in wire-hang test (**c**) of fed or 24 h-fasted 20-week-old male GRf/f (blue) and GRmKO (red) mice are shown as boxplots (*n*=15). (**d**) Endurance in forced treadmill exercise of fed or 24 h-fasted 20-week-old male GRf/f (blue) and GRmKO (red) mice are shown as latency to cease running (left panel) and travelled distance (right panel) in boxplots (*n*=10). Whiskers show the minimum and maximum of all the data, boxes show one standard deviation above and below the mean of the data, and lines inside the boxes show the median of the data (**b**–**d**). *P<0.05 determined by two-tailed Student’s *t*-test for unpaired data (**b**–**d**).

Under fasting conditions, decline in blood glucose and elevated plasma adrenocorticotropic hormone and corticosterone was comparable between GRf/f and GRmKO mice (Fig. 2a–c). Muscle messenger RNA (mRNA) expression of GR-target genes was induced in GRf/f but not in either GRmKO or adrenalectomized mice (Fig. 2d). Reduction of muscle weight was not observed in GRmKO but in GRf/f (Fig. 2e). Diurnal variation^9^ and fasting-dependent temporal elevation of plasma alanine levels were almost diminished in GRmKO mice (Fig. 2f,g). Muscle proteolysis, therefore, is transcriptionally controlled by the hypothalamus-pituitary-adrenal axis using glucocorticoid-GR, and might play a role in energy delivery to systemic circulation. Intramuscular levels of not only alanine but also pyruvate, glucose, glycogen, triglyceride and branched-chain amino acids in fasted GRmKO were significantly lower than those in GRf/f (Fig. 3a), which may coincide with severe impairment in muscle endurance and exercise capacity after fasting in GRmKO (Fig. 3b–d). Together, skeletal muscle GR appeared to play a physiological role in systemic energy supply as well as in maintenance of skeletal muscle performance.

### Enhanced lipolysis in adipose tissues of GRmKO mice

**Figure 4:**
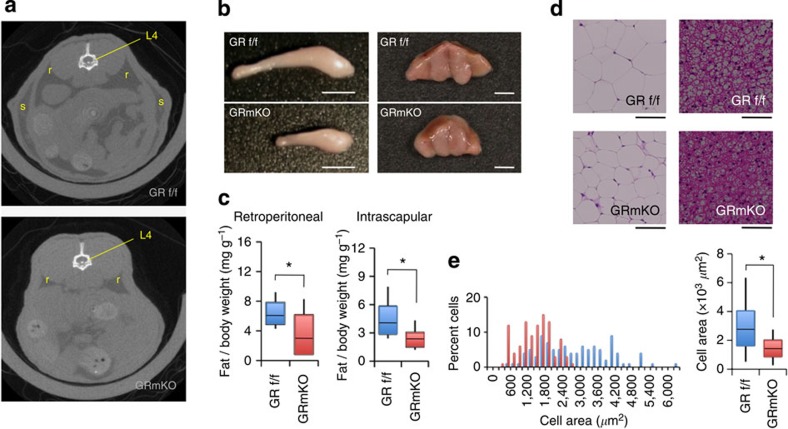
Reduced lipid storage in GRmKO mice. (**a**) Representative CT transverse section images of *ad libitum*-fed 30-week-old male GRf/f and GRmKO mice. Retroperitoneal and subcutaneous fat depots, and the 4th lumbar vertebrae are indicated as r, s and L4, respectively. (**b**,**c**) Representative photographs (**b**) and weight (**c**) of retroperitoneal fat depot (left panels, bars represent 1 cm) and intrascapular fat depot (right panels, bars represent 2 mm) from 30-week-old male GRf/f and GRmKO mice. Data are shown as boxplots of ratio to body weight (*n*=8). Whiskers show the minimum and maximum of all the data, boxes show one standard deviation above and below the mean of the data, and lines inside the boxes show the median of the data. *P<0.05 determined by two-tailed Student’s *t*-test for unpaired data. (**d**) Representative images of haematoxylin and eosin staining of retroperitoneal (left panels) and intrascapular (right panels) fat depots from 30-week-old male GRf/f and GRmKO mice. Scale bars represent 100 μm. (**e**) Adipocyte cross-sectional area (CSA) distribution of retroperitoneal fat depot from 30-week-old male GRf/f (blue) and GRmKO (red) are shown as frequency histograms (left panel, *n*=200, from three independent animals of each genotype) and boxplots (right panel). Whiskers show the minimum and maximum of all the data, boxes show one standard deviation above and below the mean of the data, and lines inside the boxes show the median of the data. *P<0.05 determined by two-tailed Student’s *t*-test for unpaired data.

**Figure 5:**
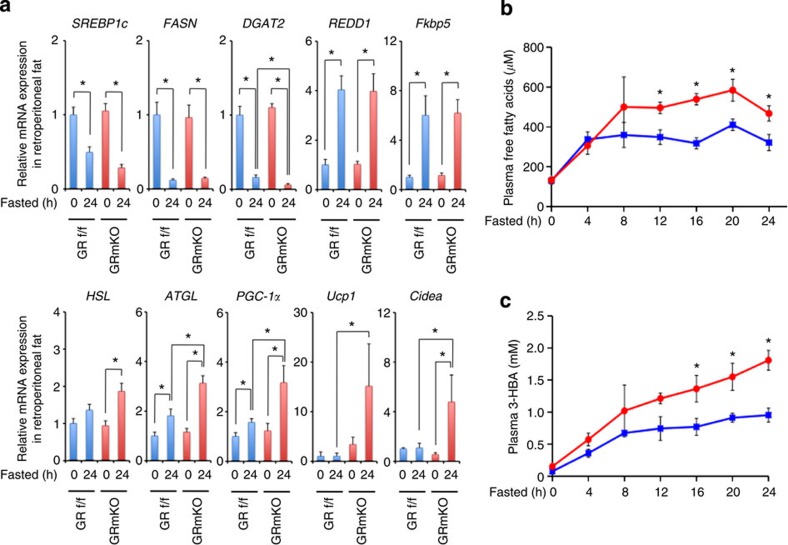
Enhanced lipolysis in adipose tissue of GRmKO mice during fasting. (**a**) Expression levels of the indicated mRNA in retroperitoneal fat from 30-week-old male GRf/f and GRmKO mice after fasting for the indicated time periods. Data are normalized to *36B4* mRNA levels and are shown as fold induction to expression levels in fed (0 h) GRf/f mice. Error bars represent mean±s.e.m. (*n*=8). *P<0.05 determined by two-tailed Student’s *t*-test for unpaired data. (**b**,**c**) Plasma concentrations of free fatty acids (**b**) and 3-hydroxybutyric acid (3-HBA, **c**) concentration of 30-week-old male GRf/f (blue) and GRmKO (red) mice after fasting for the indicated time periods. Error bars represent mean±s.e.m. (*n*=8). *P<0.05 between GRf/f and GRmKO determined by two-tailed Student’s *t*-test for unpaired data.

We, then, tried to clarify the metabolic consequence of deficient alanine supply from skeletal muscle in GRmKO mice. Of note, CT analysis indicated significant shrinkage of fat mass in GRmKO mice (Fig. 4a); the weights of retroperitoneal and intrascapular fat depots, mainly composed of white and brown adipocytes, respectively, were lower (Fig. 4b,c); and white adipocyte size was decreased in GRmKO mice (Fig. 4d,e). Fasting suppressed mRNA expression of lipogenic genes (*SREBP1c*, fatty acid synthase (*FASN*), and diacylglycerol O-acyltransferase 2 (*DGAT2*)) in white fat of both mice. Among them, *DGAT2*, a rate-limiting enzyme for triacylglycerol synthesis, was further declined in GRmKO (Fig. 5a). Although ubiquitous GR-target genes (*REDD1* and *Fkbp5*) were induced almost equally after fasting, lipolytic genes (hormone-sensitive lipase (*HSL*) and adipose triglyceride lipase (*ATGL*)), as well as several marker genes for browning of white fat peroxisome proliferator-activated receptor gamma coactivator (*PGC-1α*), uncoupling protein 1 (*Ucp1*) and *Cidea*) were further induced in GRmKO mice (Fig. 5a). Consistent with such gene expression profile, plasma free fatty acids and 3-hydroxybutyric acid were elevated in GRmKO mice after fasting (Fig. 5b,c). Fasting-induced decline in plasma insulin and C-peptide and results of intraperitoneal glucose and insulin tolerance tests were similar in those mice (Supplementary Fig. 5a–c), suggesting that glucose metabolism is not different between GRmKO mice and GR f/f mice.

### Insufficient alanine supply enhances liver *FGF21* expression

**Figure 6:**
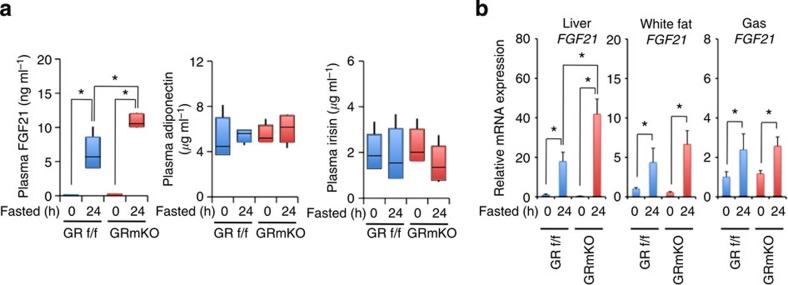
Increased systemic circulation of FGF21 in GRmKO mice during fasting. (**a**) FGF21, adiponectin and irisin concentrations in the plasma from 30-week-old male GRf/f and GRmKO mice after fasting for the indicated time periods. Data are shown as boxplots (*n*=8). Whiskers show the minimum and maximum of all the data, boxes show one standard deviation above and below the mean of the data, and lines inside the boxes show the median of the data. *P<0.05 determined by two-tailed Student’s *t*-test for unpaired data. (**b**) Expression levels of *FGF21* mRNA in the indicated tissues from 30-week-old male GRf/f and GRmKO mice after fasting for the indicated time periods. Data are normalized to *36B4* mRNA levels and are shown as fold induction to expression levels in fed (0 h) GRf/f mice. Error bars represent mean±s.e.m. (*n*=8). *P<0.05 determined by two-tailed Student’s *t*-test for unpaired data.

**Figure 7:**
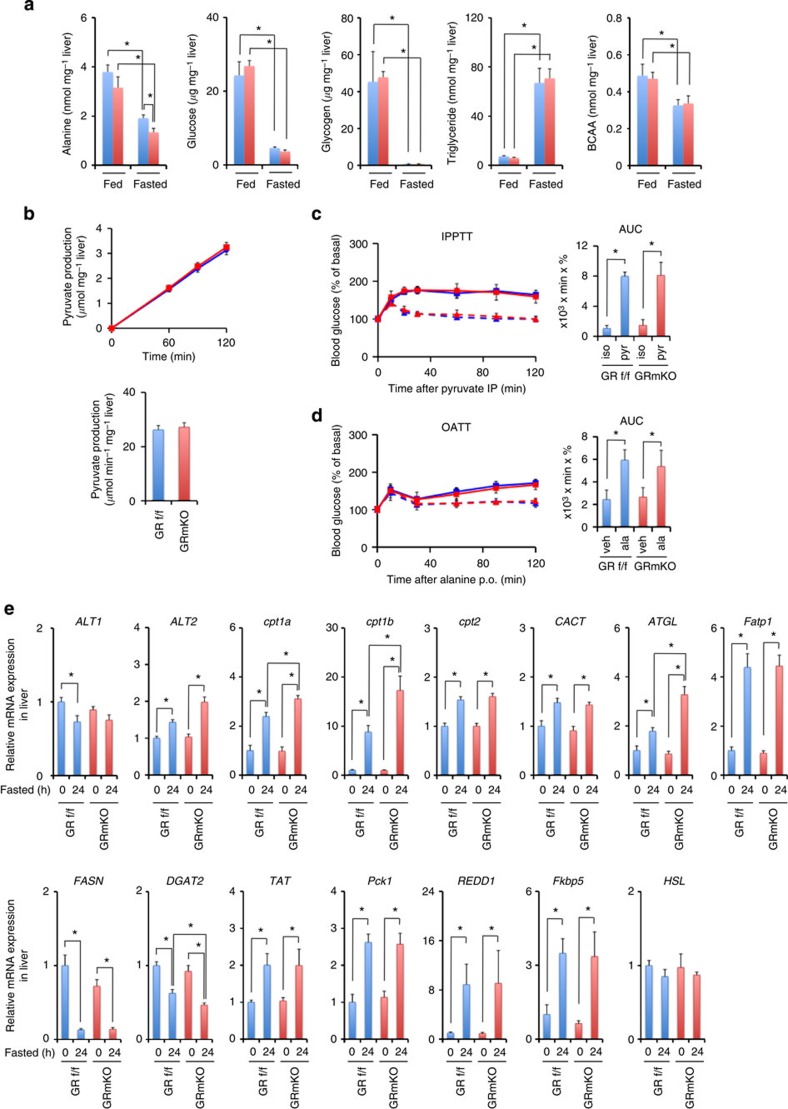
Reduced alanine content and enhanced fatty acid utilization in the liver of GRmKO mice. (**a**) Intrahepatic contents of alanine, glucose, glycogen, triglyceride and branched-chain amino acid (BCAA) in fed or 24 h-fasted 20-week-old male GRf/f (blue) and GRmKO (red) mice. Error bars represent mean±s.e.m. (*n*=6). *P<0.05 determined by two-tailed Student’s *t*-test for unpaired data. (**b**) Kinetics of alanine conversion to pyruvate by liver extracts from *ad libitum*-fed 20-week-old male GRf/f (blue) and GRmKO (red) mice. Error bars represent mean±s.e.m. (*n*=5). (**c**) Intraperitoneal pyruvate tolerance test (IPPTT) in 15-week-old male GRf/f (blue) and GRmKO (red) mice. After 4-h fasting, mice were intraperitoneally injected (i.p.) with 23 mmol kg^−1^ body weight of sodium chloride (dotted lines, iso) or sodium pyruvate (solid lines, pyr) and blood glucose levels were monitored. Time course of blood glucose clearance (left panel) and area under the curve (AUC, right panel) are shown. Error bars represent mean±s.e.m. (*n*=12). *P<0.05 determined by two-tailed Student’s *t*-test for unpaired data. (**d**) Oral alanine tolerance test (OATT) in 15-week-old male GRf/f (blue) and GRmKO (red) mice. After 4-h fasting, mice were orally administrated (p.o.) with vehicle (dotted lines, veh) or 23 mmol kg^−1^ body weight of alanine (solid lines, ala) and blood glucose levels were monitored. Time course of blood glucose clearance (left panel) and area under the curve (AUC, right panel) are shown. Error bars represent mean±s.e.m. (*n*=12). *P<0.05 determined by two-tailed Student’s *t*-test for unpaired data. (**e**) Expression levels of the indicated mRNA in the liver from 20-week-old male GRf/f and GRmKO mice after fasting for the indicated time periods. Data are normalized to *36B4* mRNA levels and are shown as fold induction to expression levels in fed (0 h) GRf/f mice. Error bars represent mean±s.e.m. (*n*=8). *P<0.05 determined by two-tailed Student’s *t*-test for unpaired data.

When we focused on serum factors that may suppress lipogenesis and stimulate lipolysis and browning of white adipose, fasting-induced elevation of plasma FGF21 appeared to be accentuated in GRmKO mice while adiponectin and irisin did not show significant differences (Fig. 6a). Fasting-induced upregulation of *FGF21* mRNA expression was prominent in the liver^10^,^11^ compared with muscle^12^ or fat^13^ in GRmKO (Fig. 6b). Concerning intrahepatic contents of energy substrates, alanine concentration was decreased in GRmKO mice in fed and fasted states (Fig. 7a), which corresponded to decreased alanine supply from skeletal muscle in GRmKO mice. *In vitro* alanine aminotransferase (ALT) activity of the liver extract and mRNA expression of *ALT1* and *ALT2* in the liver were comparable between GRmKO and GRf/f mice (Fig. 7b,e). Intraperitoneal pyruvate tolerance test (Fig. 7c) and oral alanine tolerance test (Fig. 7d) revealed similar responses in GRmKO and GRf/f mice, indicating that the balance between glucose disposal and glucose production was similarly maintained in both mice when substrates for gluconeogenesis were excessively provided. mRNA levels of genes related to fatty acid oxidation (carnitine palmitoyltransferases (*cpt1a, cpt1b*, and *cpt2*) and carnitine/acylcarnitine translocase (*CACT*)), lipolysis (*ATGL*) and fatty acid uptake (fatty acid transporter 1 (*Fatp1*)) were increased in both mice after fasting (Fig. 7e). Among them, mRNA expression of *cpt1a* and *1b*, rate-limiting enzymes for fatty acid oxidation, and *ATGL* was further enhanced in GRmKO mice (Fig. 7e). mRNA levels of lipogenic genes (*FASN* and *DGAT2*) were decreased in both mice after fasting and *DGAT2* was further declined in GRmKO mice (Fig. 7e). Several hepatic GR-target genes (tyrosine aminotransferase (*TAT*), phosphoenolpyruvate carboxykinase 1 (*Pck1*), *REDD1* and *Fkbp5*) were comparably induced after fasting (Fig. 7e), again confirming normal function of GR in the liver in both mice. *HSL* mRNA level in the liver was not significantly changed after fasting in both mice (Fig. 7e).

**Figure 8:**
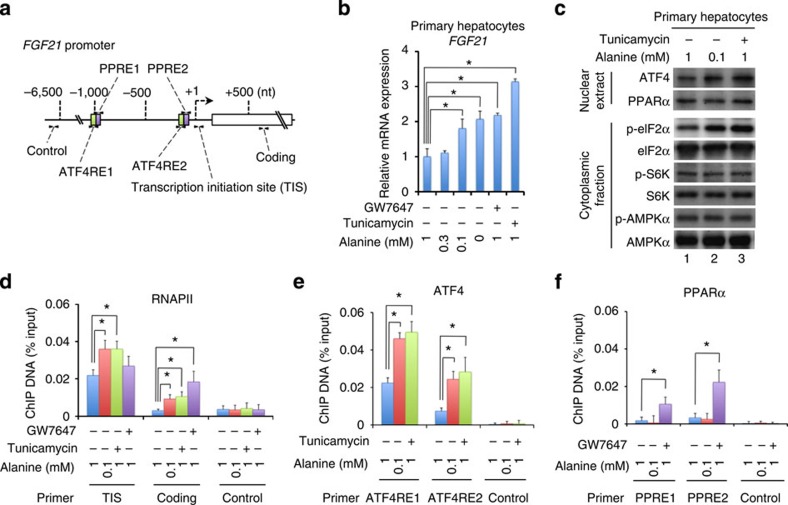
Induced *FGF21* transcription via ATF4 activation in hepatocytes exposed to alanine deprivation. (**a**) Schematic representation of mouse *FGF21* promoter. Open box, green boxes and purple boxes indicate protein-coding region, ATF4-responsive elements (ATF4RE) and PPAR-responsive elements (PPRE), respectively. Arrowheads indicate positions of primers used in chromatin immunoprecipitation (ChIP) assay (**d**–**f**). (**b**) Mouse primary hepatocytes from 10-week-old male GRf/f mice were cultured in media containing the indicated concentration of alanine along with or without 1 μM of GW7647 or 2.4 μM of tunicamycin for 5 h. Expression levels of *FGF21* mRNA were quantified with qRT–PCR analysis. Data are normalized to *36B4* mRNA levels and are shown as fold induction to expression levels in cells cultured in media containing 1 mM alanine without GW7647 or tunicamycin. Error bars represent mean±s.e.m. (*n*=3). *P<0.05 determined by two-tailed Student’s *t*-test for unpaired data. (**c**) Representative immunoblots of nuclear extracts and cytoplasmic fractions from mouse primary hepatocytes prepared and cultured as described in **b**. Uncropped data are presented in Supplementary Fig. 6. (**d**–**f**) Mouse primary hepatocytes prepared and cultured as described in **b** were subjected to ChIP using anti-RNA polymerase II (RNAPII, **d**), anti-ATF4 (**e**) or anti-PPARα (**f**) antibodies. Co-immunoprecipitated DNA fragments were quantified in real-time PCR using indicated primer pairs. Data are expressed as percentage to the DNA amounts present in the corresponding input lysates. Error bars represent mean±s.e.m. (*n*=3). *P<0.05 determined by two-tailed Student’s *t*-test for unpaired data.

Upon variety of stimuli, activating transcription factor 4 (ATF4) and peroxisome proliferator-activated receptor (PPAR) α are known to be recruited to corresponding *cis*-elements (ATF4RE and PPRE, respectively) located upstream region of *FGF21* gene^11^,^12^,^14^,^15^ (see Fig. 8a). *FGF21* mRNA expression in hepatocytes isolated from GRf/f mice was induced in response to either endoplasmic reticulum stressor tunicamycin or PPARα agonist GW7647 (Fig. 8b). When the hepatocytes were subjected to alanine deprivation, *FGF21* mRNA expression was induced (Fig. 8b). In the hepatocytes, ATF4 protein level and phosphorylation of eIF2α were increased after alanine deprivation, as seen after treatment with tunicamycin (Fig. 8c and Supplementary Fig. 6). Note that expression and phosphorylation of AMP-activated protein kinase (AMPK)-α and S6 kinase (S6K) 1 was not affected in these conditions (Fig. 8c and Supplementary Fig. 6). Chromatin immunoprecipitation (ChIP) assays revealed that alanine deprivation enhanced the recruitment of ATF4 and RNA polymerase (RNAP) II but not that of PPARα onto *FGF21* gene promoter (Fig. 8d–f). Together, decreased alanine supply, serving as a signal from muscle to liver, might accentuate *FGF21* gene transcription via activating ATF4. Subsequently, increased plasma FGF21 may act on adipose tissues and liver and stimulate fat usage.

### Exogenous alanine curtails fat consumption in GRmKO mice

**Figure 9:**
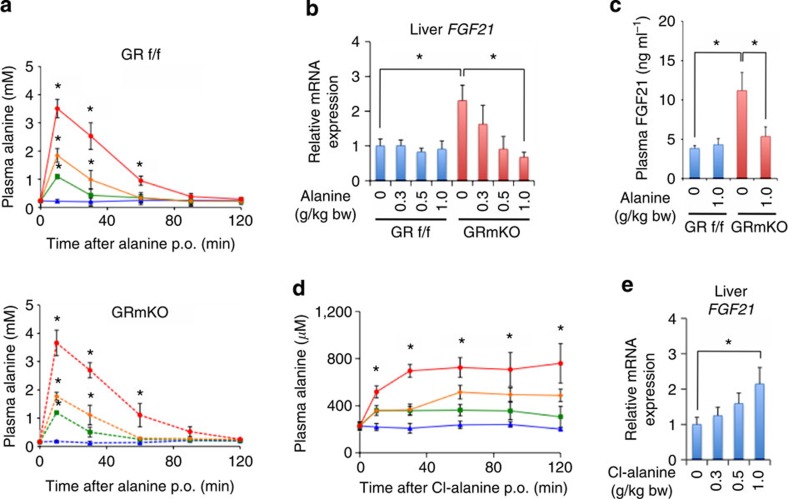
Attenuated FGF21 expression in GRmKO mice after bolus administration of alanine. (**a**) After 4-h fasting, 20-week-old male GRf/f and GRmKO mice were administrated p.o. with vehicle (blue), 0.3 (green), 0.5 (yellow) or 1.0 g kg^−1^ body weight (red) of alanine. Time course of plasma alanine levels of GRf/f (top panel) and GRmKO mice (bottom panel) are shown. Error bars represent mean±s.e.m. (*n*=4). *P<0.05 versus vehicle, determined by two-tailed Student’s *t*-test for unpaired data. (**b**) Mice were administrated alanine as described in **a** and further fasted for 24 h. Expression levels of *FGF21* mRNA in the liver are normalized to *36B4* mRNA levels and are shown as fold induction to expression levels in vehicle-treated GRf/f mice. Error bars represent mean±s.e.m. (*n*=4). *P<0.05 determined by two-tailed Student’s *t*-test for unpaired data. (**c**) Mice were administrated alanine as described in **a**, and further fasted for 24 h. Plasma FGF21 concentration are shown. Error bars represent mean±s.e.m. (*n*=4). *P<0.05 determined by two-tailed Student’s *t*-test for unpaired data. (**d**) After 4-h fasting, 20-week-old male GRf/f mice were administrated p.o. with vehicle (blue), 0.3 (green), 0.5 (yellow) or 1.0 g kg^−1^ body weight (red) of 3-chloro-L-alanine hydrochloride (Cl-alanine) and plasma alanine levels were monitored. Error bars represent mean±s.e.m. (*n*=4). *P<0.05 versus vehicle, determined by two-tailed Student’s *t*-test for unpaired data. (**e**) Mice were treated as described in **d** and further fasted for 24 h. Expression levels of *FGF21* mRNA in the liver are normalized to *36B4* mRNA levels and are shown as fold induction to expression levels in vehicle-treated mice. Error bars represent mean±s.e.m. (*n*=4). *P<0.05, determined by two-tailed Student’s *t*-test for unpaired data.

To test this scenario, we orally administered alanine to mice (Fig. 9a), and showed that hepatic mRNA expression and plasma concentrations of FGF21 were suppressed exclusively in GRmKO mice after 24 h fasting (Fig. 9b,c). Administration of an alanine analogue 3-chloro-L-alanine increased plasma alanine concentrations in GRf/f mice (Fig. 9d), most possibly reflecting the inhibition of alanine uptake and/or utilization by the liver. As expected, hepatic mRNA expression of *FGF21* after 24 h fasting was increased in a dose-dependent manner (Fig. 9e), showing a clear contrast to alanine administration (Fig. 9b). It, therefore, is strongly indicated that plasma alanine depletion and/or inhibition of alanine utilization is a key signal for modulating liver fasting response represented by increase in *FGF21* expression.

**Figure 10:**
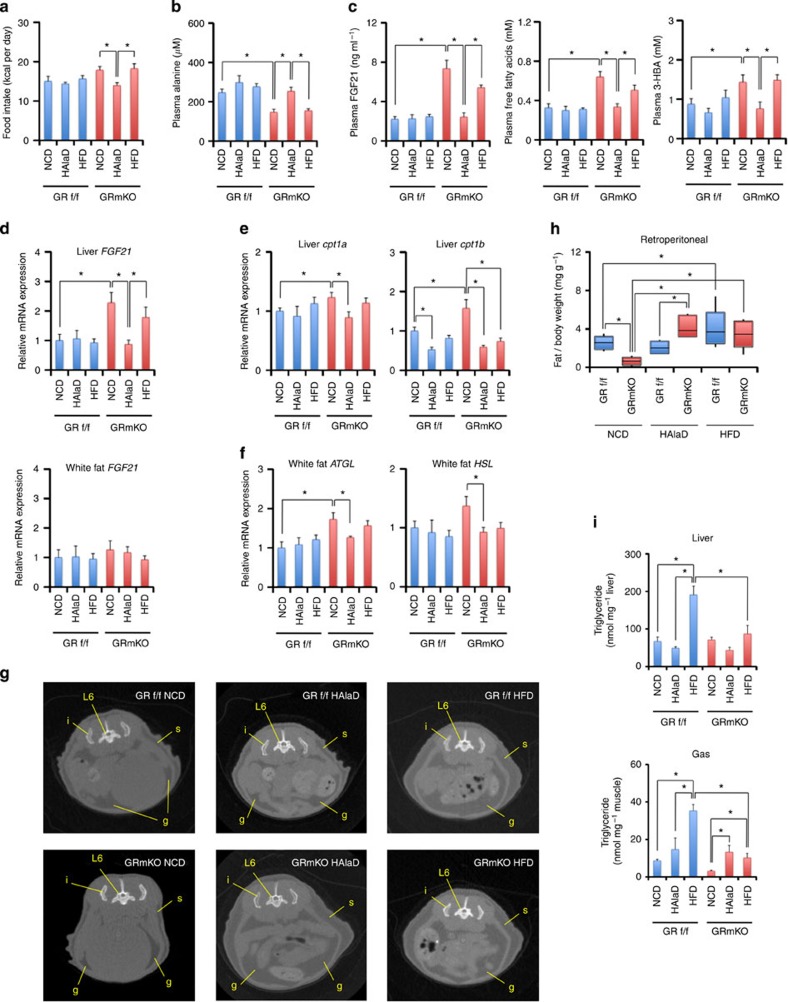
Attenuation of lipid utilization in GRmKO mice fed alanine-enriched diet. (**a**) Twelve-week-old male GRf/f and GRmKO mice were fed normal chow diet (NCD: alanine 1.4% and fat 13% of total calories), high-alanine diet (HAlaD: alanine 27% and fat 10%) or high-fat diet (HFD: alanine 1.3% and fat 32%) for 3 weeks. Food intake during this period was recorded and shown in kcal per day per individual. Error bars represent mean±s.e.m. (*n*=6). *P<0.05 determined by two-tailed Student’s *t*-test for unpaired data. (**b**) Mice were fed as described in **a**. Plasma alanine levels after 24 h-fasting are shown. Error bars represent mean±s.e.m. (*n*=6). *P<0.05 determined by two-tailed Student’s *t*-test for unpaired data. (**c**) Mice were fed as described in **a**. FGF21 (left panel), free fatty acids (middle panel) and 3-hydroxybutyric acid (3-HBA, right panel) concentrations in the plasma after 24 h-fasting are shown. Error bars represent mean±s.e.m. (*n*=6). *P<0.05 determined by two-tailed Student’s *t*-test for unpaired data. (**d**) Mice were fed as described in **a**. Expression levels of the *FGF21* mRNA in the indicated tissues from 24 h-fasted mice are normalized to *36B4* mRNA levels and are shown as fold induction to expression levels in NCD-fed GRf/f mice. Error bars represent mean±s.e.m. (*n*=6). *P<0.05 determined by two-tailed Student’s *t*-test for unpaired data. (**e**,**f**) Mice were fed as described in **a**. Expression levels of the indicated mRNA in the liver (**e**) and white fat (**f**) from 24 h-fasted mice are normalized to *36B4* mRNA levels and are shown as fold induction to expression levels in NCD-fed GRf/f mice. Error bars represent mean±s.e.m. (*n*=6). *P<0.05 determined by two-tailed Student’s *t*-test for unpaired data. (**g**) Mice were fed as described in **a**. Representative CT transverse section images are shown. Gonadal fat depots, subcutaneous fat depots, ilia and the 6th lumbar vertebrae are indicated as g, s, i and L6, respectively. (**h**) Mice were fed as described in **a**. Weights of retroperitoneal fat depots are shown as boxplots of ratio to body weight (*n*=6). Whiskers show the minimum and maximum of all the data, boxes show one standard deviation above and below the mean of the data, and lines inside the boxes show the median of the data. *P<0.05 determined by two-tailed Student’s *t*-test for unpaired data. (**i**) Mice were fed as described in **a**. Intrahepatic (top panel) and intramuscular (bottom panel) contents of triglyceride in 24 h-fasted GRf/f and GRmKO mice are shown. Error bars represent mean±s.e.m. (*n*=4). *P<0.05 determined by two-tailed Student’s *t*-test for unpaired data.

Finally, we addressed whether decreased alanine supply to the liver is a cue for systemic alteration in energy metabolism and decrease in fat mass. For that purpose, we fed those mice with normal chow diet (NCD), high-alanine diet (HAlaD) and high-fat diet (HFD) for 3 weeks. HAlaD-fed GRmKO mice showed slight decrease in food consumption (Fig. 10a), but increase in plasma alanine compared with NCD- or HFD-fed GRmKO mice (Fig. 10b). Notably, plasma FGF21, free fatty acids and 3-hydroxybutyric acid were declined exclusively in HAlaD-fed GRmKO mice after 24 h fasting (Fig. 10c). Decreased *FGF21* mRNA expression after HAlaD was not observed in white fat but in the liver (Fig. 10d). mRNA expression of *cpt1a* and *1b* in the liver and *ATGL* and *HSL* in white fat showed identical tendency with liver *FGF21* mRNA expression (Fig. 10e,f). Moreover, HAlaD restored fat mass in GRmKO as seen in HFD feeding (Fig. 10g,h). However, accumulation of triglyceride in the liver and gastrocnemius muscle caused by HFD-feeding were partially ameliorated in GRmKO (Fig. 10i), suggesting accelerated lipid consumption in GRmKO even in HFD feeding. From these results, we may conclude that shutdown of muscle-derived alanine supply to the liver is a trigger for hepatic *FGF21* induction and lipolysis in adipose tissues, and that restoration of alanine replenishes lipid deposition in GRmKO mice. Intraperitoneal glucose tolerance test revealed rather decreased glucose tolerance in GRmKO under HAlaD feeding (Supplementary Fig. 5d), indicating that adipogenic effect of HAlaD is not caused by increased glucose tolerance.

## Discussion

Muscular and lean phenotype of GRmKO mice are opposite to Cushingoid appearance characterized by central obesity and muscle atrophy seen in glucocorticoid excess^16^, strongly indicating that systemic energy distribution, at least in part, is centrally controlled via the hypothalamus–pituitary–adrenal axis and skeletal muscle GR. Glucocorticoids and skeletal muscle GR have pleiotropic roles in anabolic and catabolic regulation of carbohydrates, protein and lipid in the skeletal muscle, either directly or indirectly^17^. Decreased endurance and exercise capacity under fasting conditions in GRmKO (Fig. 3) might be due to inefficient energy production caused by depleted levels of alanine, pyruvate, glucose, glycogen and triglyceride in GR-absent-skeletal muscle (Fig. 3a), as seen in patients with glucocorticoid deficiency^18^.

We clearly showed that alanine, either directly or indirectly, transduces such a signal from muscle to liver that transcriptionally activates *FGF21* gene expression. Gluconeogenic cycle may not compensate for shortage in alanine delivery (Fig. 7b–d). Despite increased flux of free fatty acids (Fig. 5b,c), triglyceride contents in GRmKO liver are comparable to those in GRf/f liver (Fig. 7a). Moreover, HFD-feeding before fasting caused significantly lower triglyceride contents in GRmKO liver than in GRf/f liver (Fig. 10i), demonstrating facilitation of triglyceride hydrolysis and attenuation of re-esterification in GRmKO mice. This assumption is supported by mRNA profile in Fig. 7e: increased mRNA expression of *ATGL*, as well as decreased expression of *DGAT2* mRNA. Metabolic stresses including energy limitation^10^,^11^, HFD feeding^10^,^15^, dietary protein restriction^19^ and amino-acid depletion^14^,^20^, as well as endoplasmic reticulum stress^21^,^22^, mitochondrial dysfunction^12^ and cold exposure^23^, either positively or negatively, modulate *FGF21* expression in the liver. The liver, thus, integrates a variety of signals onto *FGF21* gene expression and elicits systemic regulation of energy metabolism.

In this line, adipose tissue metabolism (Fig. 4b–e) and gene expression profile (Fig. 5a) in GRmKO is largely argued by muscle-liver signalling axis-regulated FGF21 secretion, which is further supported by the effects of HAlaD (Fig. 10b–h). Lipolysis in adipose tissues is activated by multiple mechanisms including hepatic glycogen shortage^24^. Since GRmKO mice showed enhanced lipolysis despite of similar hepatic glycogen contents compared with GRf/f mice (Figs 5 and 7a), there might be an alternative cue for initiating liver-fat communication other than glycogen shortage. Although FGF21 is a possible candidate for a liver-derived messenger to decrease fat mass through activating βKlotho-FGF receptor signalling in the liver, white and brown fat^25^, and hypothalamus^26^, we cannot rule out such possibility that other molecules than FGF21, for example, other hepatokines^27^,^28^, adipokines^29^,^30^ or myokines^31^,^32^ might play a role in liver-adipose tissue communication and modulate systemic energy metabolism. In any case, further investigation including elucidation of molecular mechanisms for alanine level sensing in the liver is required for illuminating and unraveling biological processes downstream of the muscle-liver signalling.

Finally, this study clearly supports the notion that glucocorticoid-dependent alanine flux from skeletal muscle modulates hepatic metabolic profile and FGF21 production, and licenses the adipose tissue to store energy as triglyceride; plasma levels of alanine, once reaching a threshold, may decrease hepatic FGF21 production and drastically alter metabolic profile of adipose tissues to ‘thrifty’ phenotype. Of interest, hypoalaninemia is seen in pregnancy^33^, in which the shift of energy supply from muscle to fat might be beneficial for maternal activity and intrauterine child growth. Although precise mechanism remains unknown, epidemiological studies documented that plasma levels of ALT activity is closely related to future risk of obesity and diabetes^34^,^35^. Targeting the skeletal muscle-liver-fat signalling axis involving glucose-alanine cycle, therefore, would be a novel approach for treatment of patients with obesity, diabetes and metabolic syndrome.

## Methods

### Reagents

Reagents were purchased from Nacalai tesque, unless otherwise specified.

### Mice

All animal experiments were performed with the approval of the Animal Ethics Committee of the Institute of Medical Science, the University of Tokyo, the Experimental Animal Care and Use Committee of the National Institute of Neuroscience, National Center of Neurology and Psychiatry, and the Institutional Animal Care and Use Committee of Kumamoto University. B6.129P2-*Nr3c1^tm2Gsc^*/Ieg^36^ (GR-floxed mice) and B6.Cg-Tg(*ACTA1-cre*)79Jme/J^37^ (skeletal muscle actin (ACTA1)-Cre transgenic mice) were obtained from German Cancer Research Center and the Jackson Laboratory, respectively. They were crossbred to give *GR^flox/+^ACTA1-Cre^+^* (hetero GRmKO) mice. Twenty-one-day-old pups were genotyped with PCR using genomic DNA obtained from their tails, as described below (‘Genomic PCR analysis’ in this section). Regular backcrossing with C57BL/6J (CLEA Japan) was performed to prevent genetic drift. Hetero GRmKO mice were further crossbred with homozygotic *GR*-floxed (GRf/f) mice to give homozygotic skeletal muscle-specific *GR* knockout (GRmKO) mice. All mice were maintained in individual cages on a 12-h light–dark photocycle, with free access to water and a stock pellet diet (CA-1, CLEA Japan), unless otherwise stated in specific dietary studies. High alanine diet (HAlaD) was prepared by thoroughly mixing powdered CA-1 and alanine in the ratio 77:23 by weight, resulting in 20-fold enriched alanine content compared with same weight of CA-1. HFD consisted of 32% (kcal) fat was purchased from CLEA Japan (CLEA Rodent Diet Quick Fat). Eleven-week-old male GRf/f and GRmKO mice were intraperitoneally injected (i.p.) with DEX (Sigma-Aldrich) at 1 mg kg^−1^ body weight or vehicle (saline), daily for 7 days, where indicated. Alanine, 3-chloro-L-alanine hydrochloride (Tokyo Chemical Industry) or vehicle (0.5% (w/v) methyl cellulose 400cP (Wako Pure Chemical Industries) in saline) were orally administrated (p.o.) to 20-week-old male GRf/f and GRmKO mice using disposable flexible feeding needles (CL-4596 MZ-1, CLEA Japan), where indicated. Mice were anaesthetized with i.p. injection of sodium pentobarbital (Kyoritsu Seiyaku) at 75 mg kg^−1^ body weight and killed. Trunk blood was collected into heparinized sampling tubes, blood glucose levels were measured using Glucose Pilot Blood Glucose Monitoring System (Aventir Biotech) and plasma was separated and stored at −80 °C until assayed. Excised tissues were weighed, snap-frozen in isopentane cooled by liquid nitrogen and crushed using Cryo-Press (Microtec) pre-frozen in liquid nitrogen, or processed to serial 10-μm transverse cryostat sections, unless otherwise specified.

### Metabolic measurements

Twelve-week-old male GRf/f and GRmKO mice were analysed. Basal locomotor activity during 24 h was measured using ACTIMO-S food intake, drinking and locomotor activity monitoring system (Shintechno). Body temperature was measured using a rectal probe attached to a digital thermometer (Thermalert TH-5, Physitemp). Oxygen consumption (VO_2_), carbon dioxide production (VCO_2_) and respiratory exchange ratio (RER) were measured every 5 min over 24 h under resting condition using an O_2_/CO_2_ metabolism measuring system for small animals (MK-5000RQ, Muromachi Kikai). Energy expenditure was calculated from the gas exchange data [energy expenditure=(3.815+1.232 × RER) × VO_2_].

### Genomic PCR analysis

Genomic DNA was extracted from tail tip biopsies (only from 21-day-old pups) or crushed tissues using Wizard Genomic DNA Purification Kit (Promega). PCR was performed with KOD FX (TOYOBO). Amplified DNA fragments were electrophoretically resolved in 2% agarose gel containing 5 mg l^−1^ of ethidium bromide and visualized using ultraviolet image Analyzer FAS-IV system (Nippon Genetics). Sequences of primers used in genomic PCR analysis are listed in Supplementary Table 1.

### Mouse primary hepatocytes

Ten-week-old male GRf/f mice were anaesthetized with i.p. injection of sodium pentobarbital at 50 mg kg^−1^ body weight. Liver was perfused with 40 ml of Liver Perfusion Medium (Life Technologies) supplemented with 100 U ml^−1^ penicillin and 100 μg ml^−1^ streptomycin (Life Technologies), and then further perfused with 80 ml of Liver Digest Medium (Life Technologies). Hepatocytes were recovered from the digested liver, passed through 100-μm nylon mesh cell strainer (Corning Inc) and washed twice with ice-cold Hepatocyte Wash Medium (Life Technologies) supplemented with 50 U ml^−1^ penicillin and 50 μg ml^−1^ streptomycin. Hepatocytes were suspended in William's E Medium Glutamax Supplement (Life Technologies) supplemented with 5% fetal bovine serum (Life Technologies), 50 U ml^−1^ penicillin, and 50 μg ml^−1^ streptomycin and plated onto collagen I-coated culture dish (AGC Techno Glass Co., Ltd.) at a density of 5 × 10^4^ cells per cm^2^. Cells were cultured in a humidified atmosphere at 37 °C with 5% CO_2_ for 4 h, culture media were replaced to fresh media and cells were further cultured for 16 h. Cells were washed with PBS twice and culture media were replaced to Dulbecco’s modified Eagle’s medium (no glucose) with L-Gln, without sodium pyruvate supplemented with 0.1% fetal bovine serum, 100 nM DEX, 50 U ml^−1^ penicillin and 50 μg ml^−1^ streptomycin. Cells were treated with 0.1–1 mM alanine, 2.4 μM tunicamycin (Calbiochem) or 1 μM GW7647 (Cayman Chemical Company) for 5 h and subjected to biochemical analyses.

### Quantitative reverse transcription PCR (qRT–PCR) analysis

Total RNA was extracted from crushed tissues or cell pellet using Sepasol-RNA I Super G and subjected to reverse-transcription with oligo-dT primer (Invitrogen) using SuperScript III First-Strand Synthesis System for RT–PCR (Invitrogen). PCR was performed with THUNDERBIRD Probe qPCR Mix or SYBR qPCR Mix (TOYOBO), Universal ProbeLibrary Sets (Roche) and CFX96 Real-Time PCR Detection System (Bio-Rad). Expression levels of mRNA were calculated on the basis of standard curves generated for each primer pair and *36B4* mRNA was used as an invariant control. Sequences of primers used in qRT–PCR analysis are listed in Supplementary Table 1.

### Immunoblotting

Crushed tissues were lysed in RIPA buffer (50 mM tris(hydroxymethyl)aminomethane, pH 7.6; 150 mM sodium chloride; 1% Nonidet P-40; 0.5% sodium deoxycholate (Sigma-Aldrich); 0.1% sodium dodecyl sulfate) supplemented with 1 mM dithiothreitol, 100 nM MG132 (Sigma-Aldrich), Protease Inhibitor Cocktail for Use with mammalian Cell and Tissue extracts (25955-11) and Phosphatase Inhibitor Cocktail (06863-01). Soluble fractions were collected and used as whole tissue extracts. NE-PER Nuclear and Cytoplasmic Extraction Reagents (Thermo Fisher Scientific Inc.) was used to prepare nuclear extracts and cytoplasmic fractions from cultured cells. Protein concentration of each sample was measured using BCA protein assay kit (Thermo Fisher Scientific Inc.). Electrophoretically resolved proteins were transferred onto polyvinylidene difluoride membranes (Millipore) and blotted with antibodies listed in Supplementary Table 2. Proteins were visualized and quantified using horseradish peroxidase-linked secondary antibodies (GE Healthcare), Signal Enhancer HIKARI, Chemi-Lumi One Super and Luminoimage Analyzer LAS-1000mini (Fujifilm).

### ChIP assay

Cells were cross-linked with 1% formaldehyde in PBS for 10 min at 37 °C. Cross-linking was stopped with addition of glycine to a final concentration of 125 mM and subsequent incubation for 5 min at 37 °C. Cells were rinsed with ice-cold PBS twice and resuspended in buffer S (50 mM tris(hydroxymethyl)aminomethane, pH 8.0; 1% sodium dodecyl sulfate; 10 mM ethylenediaminetetraacetic acid) supplemented with 1 mM dithiothreitol, 100 nM MG132, Protease Inhibitor Cocktail for Use with mammalian Cell and Tissue extracts and Phosphatase Inhibitor Cocktail, and incubated at 10 °C for 10 min. Samples were sheared to an average size of 500 bp by sonication. Lysates corresponding to 3.5 × 10^6^ cells were diluted tenfold in buffer D (0.01% sodium dodecyl sulfate; 1.1% Triton X-100 (Sigma-Aldrich); 1.2 mM ethylenediaminetetraacetic acid; 16.7 mM tris(hydroxymethyl)aminomethane, pH 8.0; 167 mM sodium chloride) supplemented with 100 nM MG132, Protease Inhibitor Cocktail for Use with mammalian Cell and Tissue extracts and Phosphatase Inhibitor Cocktail, and precleared with Protein A agarose/salmon sperm DNA (Millipore) at 4 °C for 30 min. Supernatants were incubated with antibodies listed in Supplementary Table 2 at 4 °C for 18 h. Protein A agarose/salmon sperm DNA was added and further incubated at 4 °C for 1 h. Beads were washed twice each with Buffer W1 (0.1% sodium dodecyl sulfate; 1% Triton X-100; 2 mM ethylenediaminetetraacetic acid; 20 mM tris(hydroxymethyl)aminomethane, pH 8.0; 150 mM sodium chloride), Buffer W2 (0.1% sodium dodecyl sulfate; 1% Triton X-100; 2 mM ethylenediaminetetraacetic acid; 20 mM tris(hydroxymethyl)aminomethane, pH 8.0; 500 mM sodium chloride), Buffer W3 (0.25 M lithium chloride; 1% Nonidet P-40; 1% sodium deoxycholate; 1 mM ethylenediaminetetraacetic acid; 10 mM tris(hydroxymethyl)aminomethane, pH 8.0) and Buffer W4 (10 mM tris(hydroxymethyl)aminomethane, pH 8.0; 1 mM ethylenediaminetetraacetic acid). Protein–chromatin complex was eluted with elution buffer (10 mM dithiothreitol, 1% sodium dodecyl sulfate, 0.1 M sodium hydrogen carbonate). Eluates and input lysates were reverse cross-linked by incubation in 200 mM sodium chloride at 65 °C for 16 h. Proteins in the samples were digested with 36 ng μl^−1^ proteinase K at 45 °C for 1 h, and DNA fragments were recovered using QIAquick DNA purification kit (Qiagen). The amounts of DNA fragments in the eluates and in the input lysates were quantified with real-time PCR using SYBR qPCR Mix and CFX96 Real-Time PCR Detection System on the basis of standard curves generated for each primer pair. Results were calculated as percentage of ChIP DNA to input. Sequences of primers used in ChIP assays are listed in Supplementary Table 1.

### Indirect immunofluorescent staining and fluorescence imaging

Muscle cryosections were blocked with 5% goat serum/1% BSA in PBS and incubated with antibodies listed in Supplementary Table 2. After washing with PBS, specimens were incubated with secondary antibodies labelled with Alexa Fluor 488 or Alexa Fluor 568 (Invitrogen, 1:1,000) and mounted in VECTASHIELD Mounting Medium for Fluorescence with DAPI (Vector Laboratories). Images of the immunofluorescent staining were recorded using fluorescence microscope Biozero BZ-8000 system (KEYENCE). Fibre size was quantified using Image J software (National Institutes of Health).

### Computed tomography

Fifteen-, 18- or 30-week-old male GRf/f and GRmKO mice were anaesthetized with isoflurane (DS Pharma Animal Health), and analysed using *in vivo* micro X-ray CT system R_mCT2 (Rigaku).

### Plasma biochemistry

Plasma concentrations of adrenocorticotropic hormone (ACTH, EK-001-21, Phoenix Pharmaceuticals), corticosterone (YK240, Yanaihara Institute) and irisin (EK-067-16, Phoenix Pharmaceuticals) were measured by enzyme immunoassays using iMark Microplate Absorbance Reader (Bio-Rad), according to the manufacturer’s instructions. Plasma concentrations of insulin (MS303, Morinaga Institute of Biological Science), C-peptide (AKRCP-031, Shibayagi), FGF21 (MF2100, R&D Systems) and adiponectin (AKMAN-011, Shibayagi) were measured by ELISA using iMark Microplate Absorbance Reader, according to the manufacturer’s instructions. Plasma concentration of alanine (K652-100, BioVision), free fatty acids (K612-100, BioVision) and 3-hydroxybutyric acid (Ketone Test B ‘Sanwa’ Liquid, Sanwa Kagaku Kenkyusho) were measured by colorimetric assays using NanoDrop 2000c (Thermo Fisher Scientific Inc.), according to the manufacturer’s instructions.

### Quantification of metabolites in mouse tissues

Quantification of alanine and pyruvate (K652-100, BioVision), free fatty acids (K612-100, BioVision), glucose and glycogen (K646-100, BioVision), triglyceride (K622-100, BioVision) and branched-chain amino acids (BCAA, K564-100, BioVision) were measured by colorimetric assays using NanoDrop 2000c according to manufacturer’s instructions.

### Measurement of ALT activity

ALT activity in liver extract was measured by colorimetric assay (K752-100, BioVision), using iMark Microplate Absorbance Reader, according to the manufacturer’s instructions.

### Adrenalectomy

Nineteen-week-old male C57BL/6J mice were anaesthetized with i.p. injection of sodium pentobarbital at 50 mg kg^−1^ body weight. A 0.5-cm skin incision was made in the back, the skin on both sides of the incision was moved to the side and a 2-mm posterior abdominal wall incision was made on the top of each adrenal gland. The entire adrenal gland was clamped for 1 min to stop the blood flow, and then extirpated from the body using a pair of sterile ring forceps (A-26, Natsume Seisakusho). The skin incision was closed with surgical suture (ER12-50B2, Natsume Seisakusho). The mice were placed into individual clean cages and recovered on a 35 °C heater mat (KN-475, Natsume Seisakusho). After recovery, the mice were maintained by providing food and drinking water containing 0.9% sodium chloride *ad libitum*. Experiments were performed 7 days after adrenalectomy.

### Measurement of grip strength

Grip strength of 20-week-old male GRf/f and GRmKO mice was measured with MK-380CM/R (Muromachi Kikai). After mouse’s forelimbs and hindlimbs were placed on a mesh, the mouse was gently pulled back until it lost its grip from the mesh. The maximal force generated at the point where the animal loses its grip was measured in grams of resistance by a strain gauge. The highest score among five trials with 30-s recovery period between each trial was recorded.

### Passive wire-hang test

Twenty-week-old male GRf/f and GRmKO mice were placed on the cage top, which was then inverted and suspended approximately 35-cm above the home cage covered with stretchable cloths as cushions. The latency to when the animal falls was measured. If mice managed to hang face down by grabbing the cage with their hindlimbs just before the fall, the mice were considered to have jumped off on purpose and the scores were ignored. If the latency period reached 75 s, the animal was given a score 75 s. The highest score among three trials with 30-s recovery period between each trial was recorded.

### Treadmill exercise

Treadmill exercise test was performed using MK-680AT/02M (Muromachi Kikai). Twenty-week-old male GRf/f and GRmKO mice were warmed up at a speed of 5 m min^−1^ for 1 min. Every subsequent 1 min, the speed was increased by 2 m min^−1^ towards a maximum speed of 15 m min^−1^. Then, mice ran for total 45 min or until exhaustion. Exhaustion was defined as the inability to continue regular treadmill running despite repeated electric prodding to the mice. Running time was measured and running distance was calculated.

### Assays for glucose homeostasis

For glucose tolerance test (IPGTT), 15- or 30-week-old male GRf/f and GRmKO mice were fasted for 16 h with free access to water. Glucose (1 g kg^−1^ body weight) was administrated i.p. and blood glucose levels in tail vein blood were measured at 0, 10, 20, 30, 60, 90 and 120 min, using Glucose Pilot Blood Glucose Monitoring System. For insulin tolerance test (IPITT), 30-week-old male GRf/f and GRmKO mice were fasted for 4 h with free access to water. Insulin (0.75 U kg^−1^ body weight) was administrated i.p. and blood glucose levels in tail vein blood were measured at 0, 30, 60, 90 and 120 min. For pyruvate tolerance test (IPPTT), 15-week-old male GRf/f and GRmKO mice were fasted for 4 h with free access to water. Sodium pyruvate (23 mmol kg^−1^ body weight) or sodium chloride (23 mmol kg^−1^ body weight, as an isoionic control) was administrated i.p. and blood glucose levels in tail vein blood were measured at 0, 10, 20, 30, 60, 90 and 120 min. For oral alanine tolerance test, 15-week-old male GRf/f and GRmKO mice were fasted for 4 h with free access to water. Alanine (23 mmol kg^−1^ body weight) were orally administrated (p.o.) as described above (‘Mice’ in this section), and blood glucose levels in tail vein blood were measured at 0, 10, 20, 30, 60, 90 and 120 min.

### Statistical analysis

Data were analysed with two-tailed Student’s *t*-test for unpaired data. *P* values below 0.05 were considered statistically significant. Bar graphs and line graphs show means±standard error of the mean (s.e.m.). In boxplot graphs, whiskers show the minimum and maximum of all the data, a box shows one standard deviation above and below the mean of the data, and a line inside the box shows the median of the data.

## Author contributions

N.S., N.I., S.T. and H.T. designed the research. N.S., T.M., A.K.-S., R.M., N.I., N.Y., K.M., Y.O., Y.T. and Y.M. performed the experiments. S.B. and G.S. provided essential materials and made comments on the manuscript. N.S. and H.T. wrote the manuscript. H.T. supervised the research.

## Additional information

**How to cite this article:** Shimizu, N. *et al.* A muscle-liver-fat signalling axis is essential for central control of adaptive adipose remodelling. *Nat. Commun.* 6:6693 doi: 10.1038/ncomms7693 (2015).

## Supplementary Material

Supplementary Information Supplementary Figures 1-6 and Supplementary Tables 1-2
