# Supplementary material for: A muscle-liver-fat signalling axis is essential for central control of adaptive adipose remodelling
Source: Nat Commun. 2015 Apr 1;6:6693. doi: 10.1038/ncomms7693 (PMC4396397; doi:10.1038/ncomms7693)
Supplement: Supplementary Information — Supplementary Figures 1-6 and Supplementary Tables 1-2 [file ncomms7693-s1.pdf]

Supplementary Figure 1

N. Shimizu, et al.

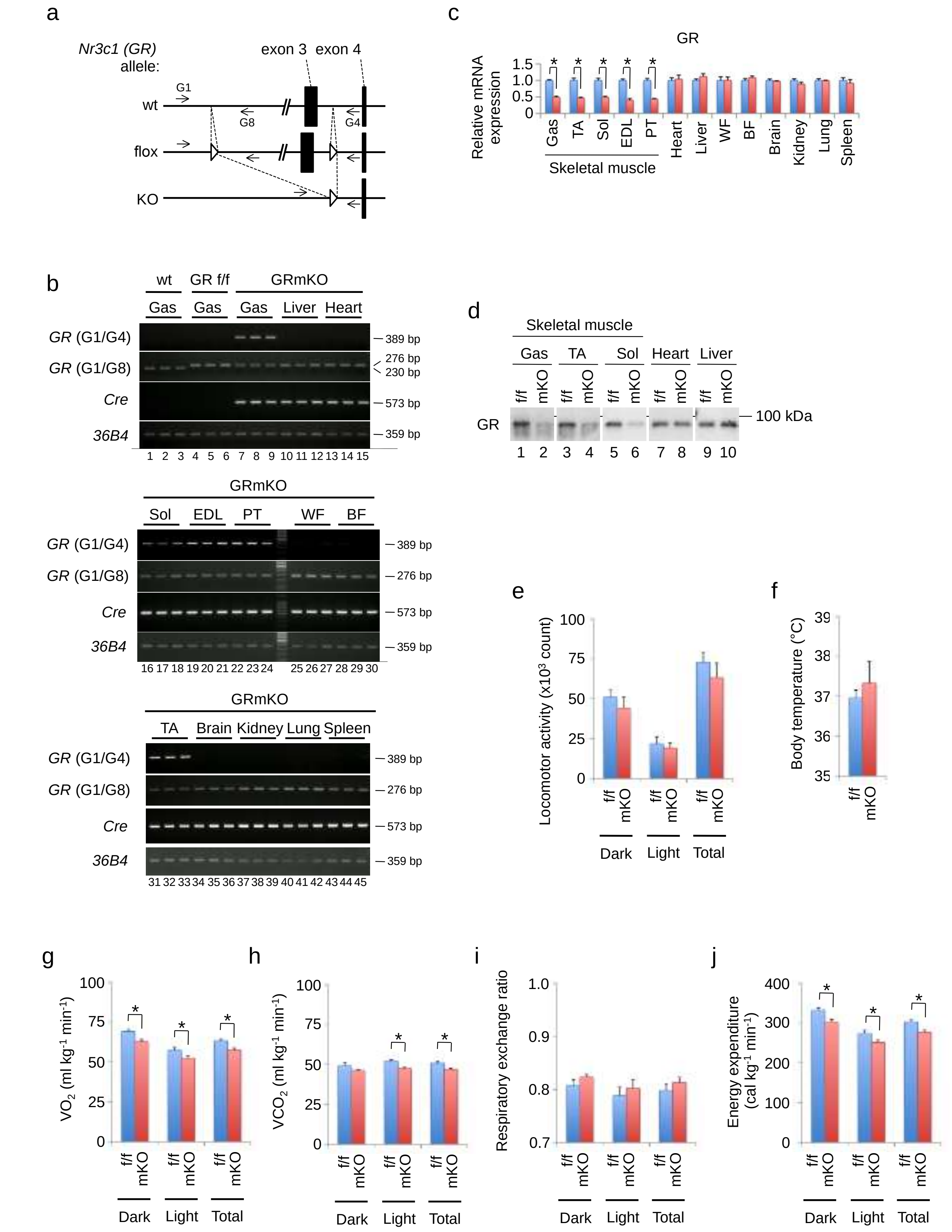

Supplementary Figure 1

Whole body metabolic profiles of skeletal muscle-specific GR knockout mice.

(**a**) Nr3c1 (GR) allele including exon 3 and 4 of wild type C57BL/6J (wt), Nr3c1^{tm2Gsc} (flox) [36], and a consequence of Cre-mediated recombination (KO) are schematically depicted. Open triangles indicate loxP sites. Arrows indicate primers used for genomic PCR.

(**b**) Skeletal muscle actin (ACTA1)-Cre transgenic mice[37] and GR-floxed (GRf/f) mice were crossbred to give skeletal muscle-specific GR knockout (GRmKO) mice, as described in Methods. Genomic DNA from the indicated tissues of 10-week-old male wt, GRf/f, and GRmKO mice were analyzed with PCR to confirm skeletal muscle-specific recombination of GR allele. Data from three independent animals of each genotype are shown. Skeletal muscles and adipose tissues are abbreviated as Gas (gastrocnemius), TA (tibialis anterior), Sol (soleus), EDL (extensor digitorum longus), PT (plantaris), WF (retroperitoneal fat depot as white fat), and BF (intrascapular fat depot as brown fat).

(**c**) Expression levels of GR mRNA in the indicated tissues from 10-week-old male GRf/f (blue) and GRmKO (red) mice. Data from quantitative reverse transcription PCR (qRT-PCR) analysis are normalized to 36B4 mRNA levels and are shown as fold induction to expression levels in GRf/f mice. Error bars represent mean ± SEM (n = 8). *P < 0.05 determined by two-tailed Student's t test for unpaired data.

(**d**) Expression levels of GR protein in the indicated tissues from 10-week-old male GRf/f and GRmKO mice. Total tissue extracts were analyzed in immunoblotting, and representative data from 3 independent animals are shown.

(**e**) Basal locomotor activity of 12-week-old male GRf/f and GRmKO mice during 12 h in the dark phase [zeitgeber time (ZT) 12-24], during 12 h in the light phase (ZT 0-12), and during 24 h of a whole day (Total, ZT 0-24). Error bars represent mean ± SEM [n = 5 (GRf/f) and n = 4 (GRmKO)].

(**f**) Body temperature of 12-week-old male GRf/f and GRmKO mice. Error bars represent mean ± SEM [n = 5 (GRf/f) and n = 4 (GRmKO)].

(**g**-**j**) Resting oxygen consumption rates (**g**), carbon dioxide production rates (**h**), respiratory exchange ratios (**i**), and energy expenditures (**j**) during the time periods described in (**e**). Error bars represent mean ± SEM [n = 5 (GRf/f) and n = 4 (GRmKO)]. *P < 0.05 determined by two-tailed Student's t test for unpaired data.

Supplementary Figure 2

N. Shimizu, et al.

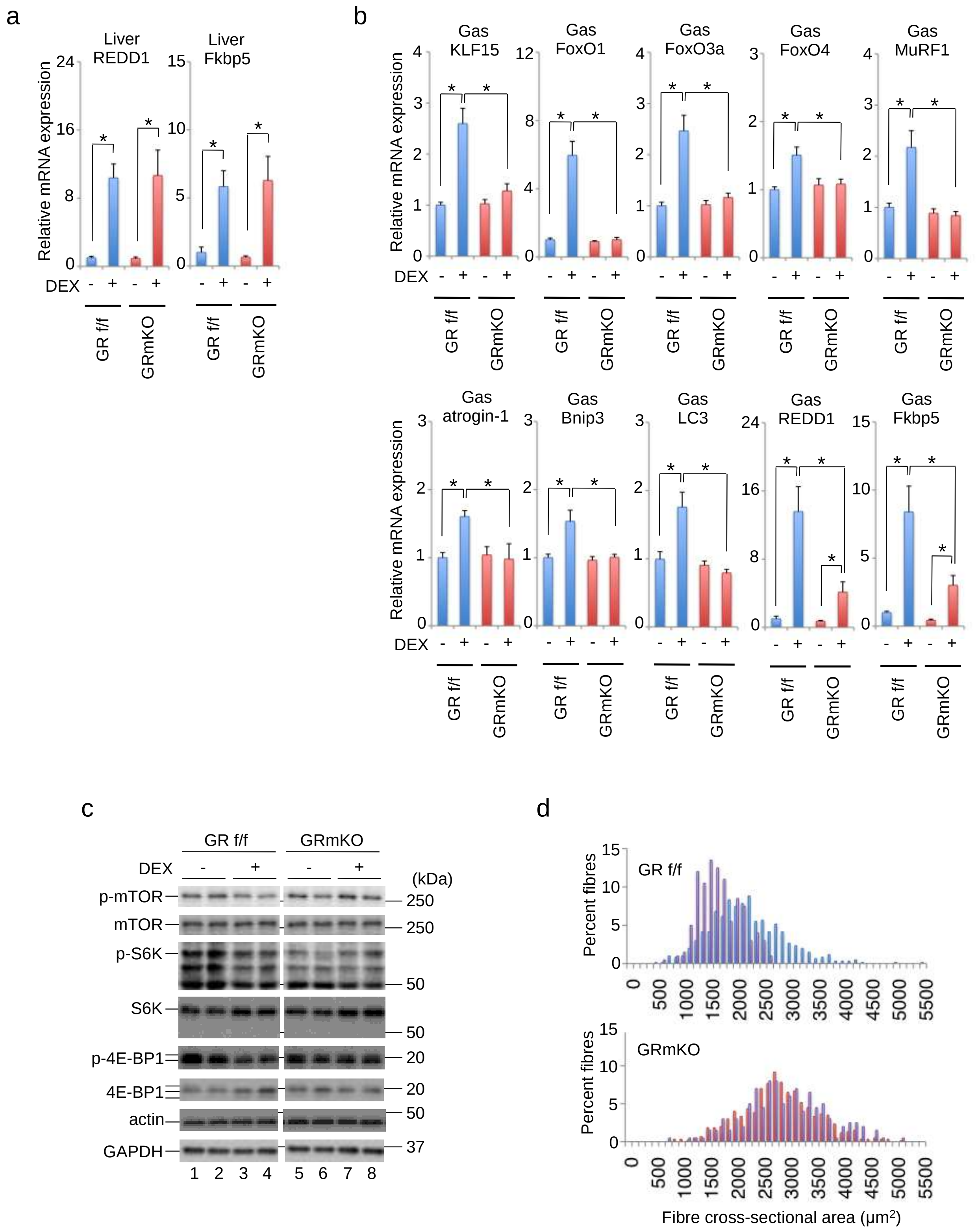

Supplementary Figure 2

Restoration of GRmKO skeletal muscle from glucocorticoid-induced atrophy.

(**a, b**) Eleven-week-old male GRf/f and GRmKO mice were injected i.p. with vehicle or dexamethasone (DEX) at 1 mg/kg body weight daily for 7 days. Expression levels of the mRNA in liver (**a**) and Gas (**b**) were assessed with qRT-PCR. Data are normalized to 36B4 mRNA levels and are shown as fold induction to expression levels in vehicle-treated GRf/f mice. Error bars represent mean ± SEM (n = 8). *P < 0.05 determined by two-tailed Student's t test for unpaired data.

(**c**) Expression and phosphorylation levels of the indicated proteins in Gas from male GRf/f and GRmKO mice treated as described in (**a**) were assessed in immunoblotting. Data from 2 independent animals of each treatment and genotype are shown.

(**d**) Eleven-week-old male GRf/f and GRmKO mice were treated with vehicle or DEX as described in (**a**). Cross-sectional areas (CSA) of myofibres in vehicle-treated GRf/f (top panel, blue), DEX-treated GRf/f (top panel, purple), vehicle-treated GRmKO (bottom panel, red), and DEX-treated GRmKO (bottom panel, purple) are quantified (200 < n < 250, from 3 independent animals of each treatment and genotype) and their distributions in size are shown as frequency histograms.

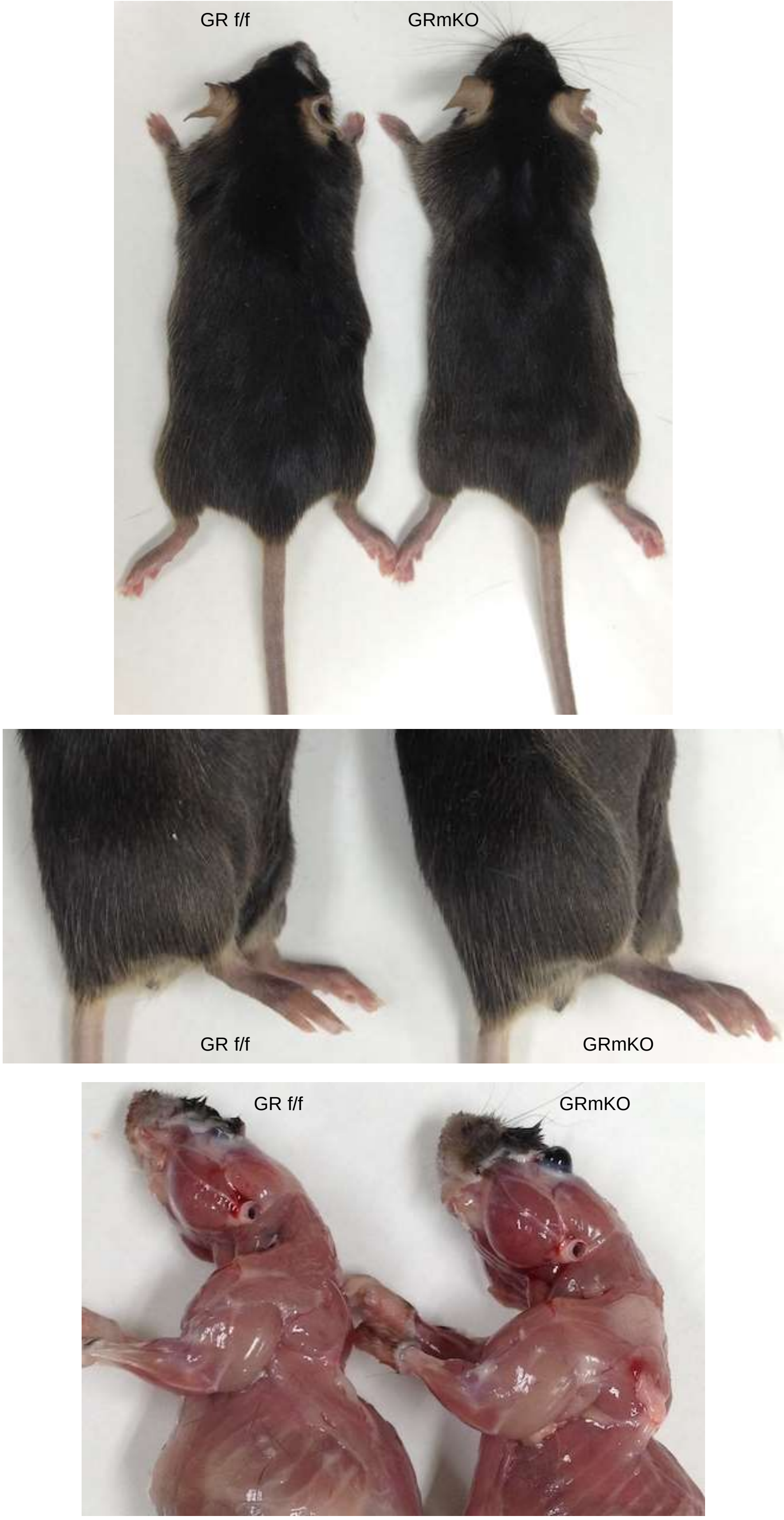

Supplementary Figure 3
Gross appearance of GRf/f and GRmKO mice.
Representative photographs of 18-week-old male GRf/f and GRmKO mice.

Supplementary Figure 4

N. Shimizu, et al.

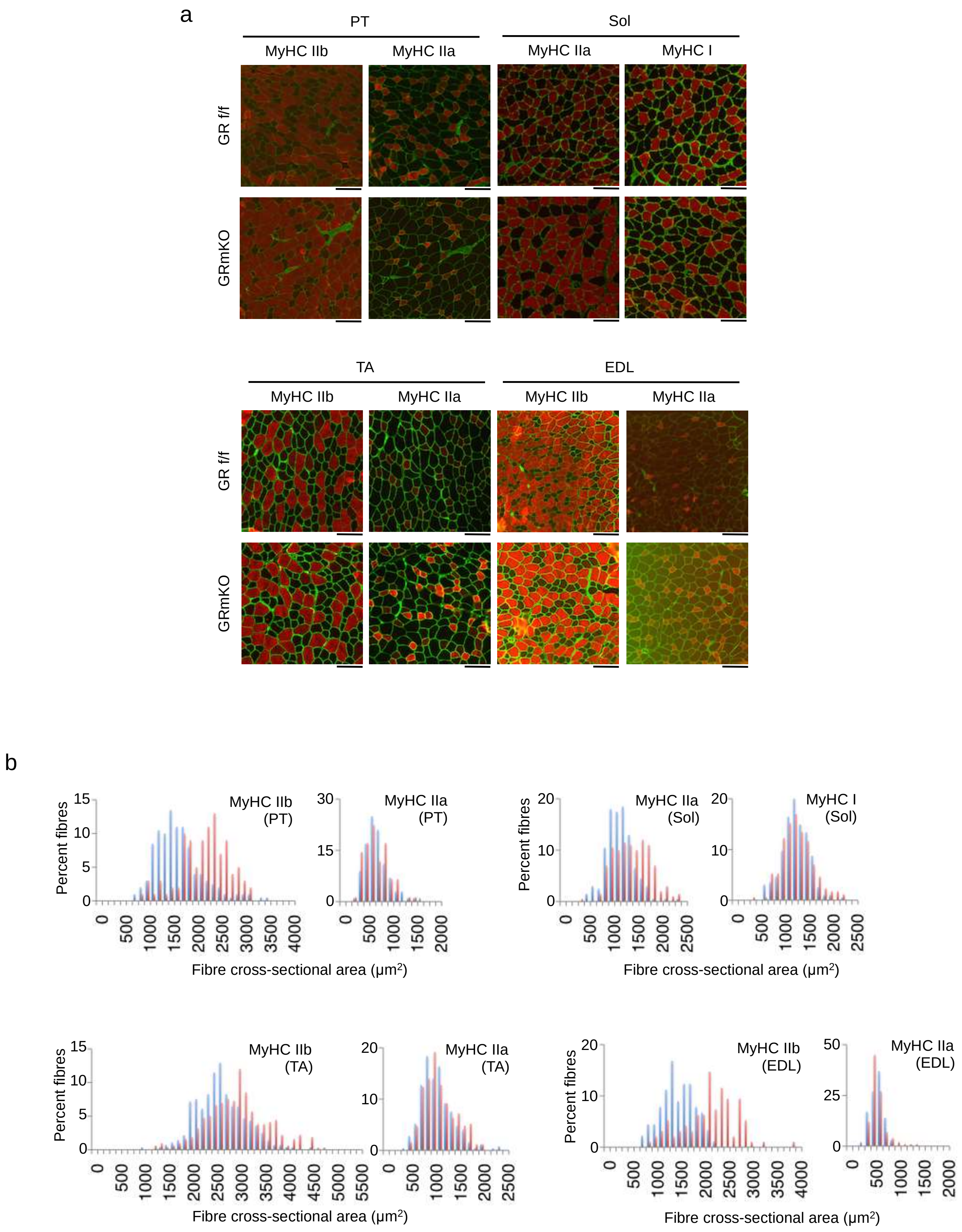

Supplementary Figure 4

Fibre type-specific hypertrophy in GRmKO mice skeletal muscle.

(**a**) Representative images of immunostaining for type IIb, IIa, and I myosin heavy chain (red in the panels indicated as MyHC IIb, MyHC IIa, and MyHC I, respectively) and type IV collagen (green) of serial transverse cryosections of indicated muscles from 20-week-old male GRf/f and GRmKO mice. Bars represent 100 μm.

(**b**) Myofibre CSA distribution in the indicated muscles from 20-week-old male GRf/f (blue) and GRmKO (red) are shown as frequency histograms ($300 < n < 330$, from 3 independent animals of each genotype).

Supplementary Figure 5

N. Shimizu, et al.

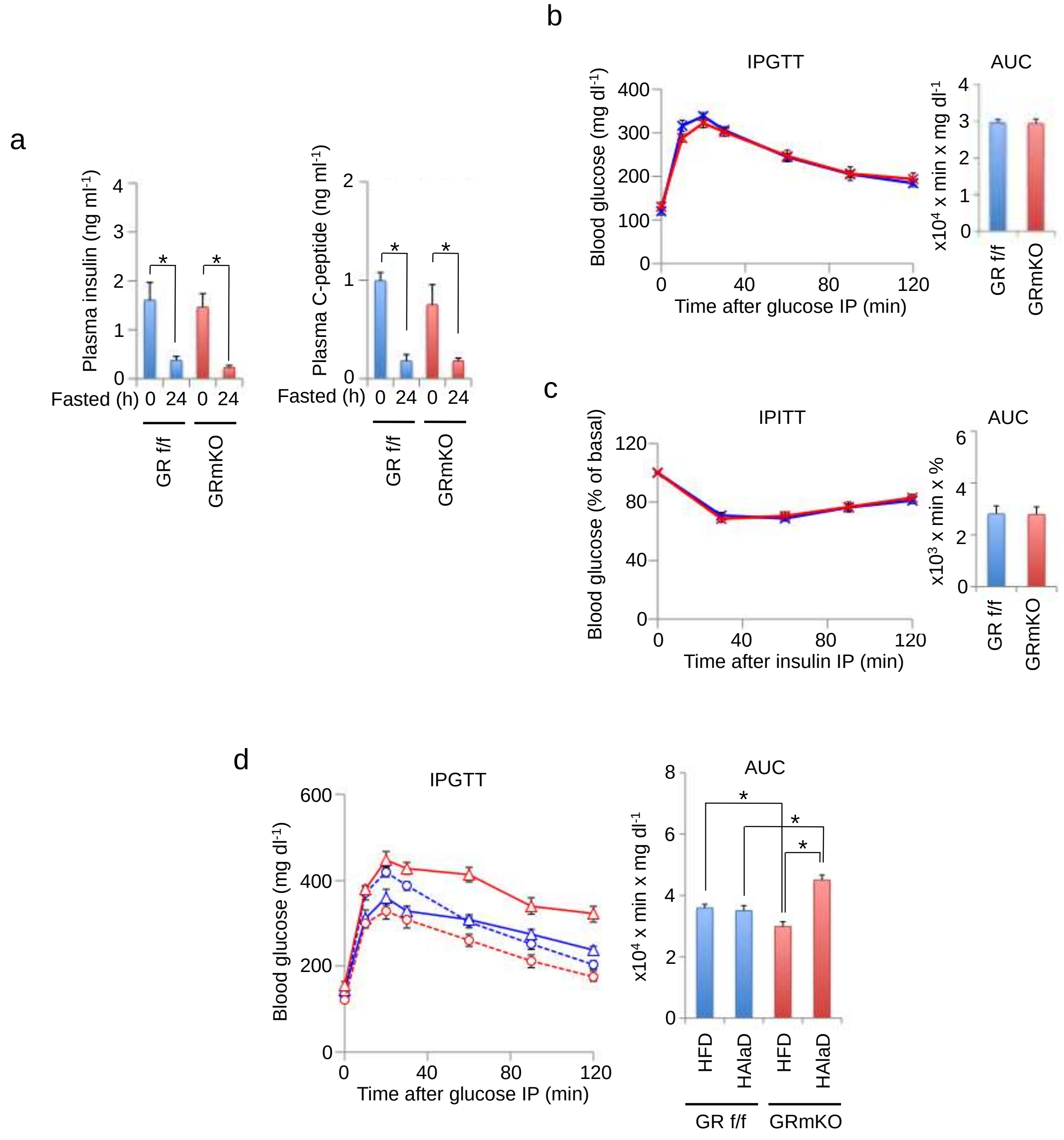

Supplementary Figure 5

Plasma insulin and C-peptide levels and glucose homeostasis in GRf/f and GRmKO mice.

(**a**) Plasma insulin (left panel) and C-peptide (right panel) levels of 30-week-old male GRf/f and GRmKO mice after fasting for the indicated time periods. Error bars represent mean ± SEM (n = 8). *$P < 0.05$ determined by two-tailed Student's t test for unpaired data.

(**b**) Intraperitoneal glucose tolerance test (IPGTT) in 30-week-old male GRf/f (blue) and GRmKO (red) mice. Time course of blood glucose clearance (left panel) and area under the curve (AUC, right panel) are shown. Error bars represent mean ± SEM (n = 12).

(**c**) Intraperitoneal insulin tolerance test (IPITT) in 30-week-old male GRf/f (blue) and GRmKO (red) mice. Time course of blood glucose clearance (left panel) and AUC (right panel) are shown. Error bars represent mean ± SEM (n = 12).

(**d**) IPGTT in 15-week-old male GRf/f and GRmKO mice fed as described in the legend for **Fig. 10a**. Time course of blood glucose clearance (left panel) and AUC (right panel) of GRf/f (blue) and GRmKO (red) mice are shown. Solid lines and dotted lines correspond HAlaD- and HFD-fed mice, respectively. Error bars represent mean ± SEM (n = 12). *$P < 0.05$ determined by two-tailed Student's t test for unpaired data.

Supplementary Figure 6

N. Shimizu, et al.

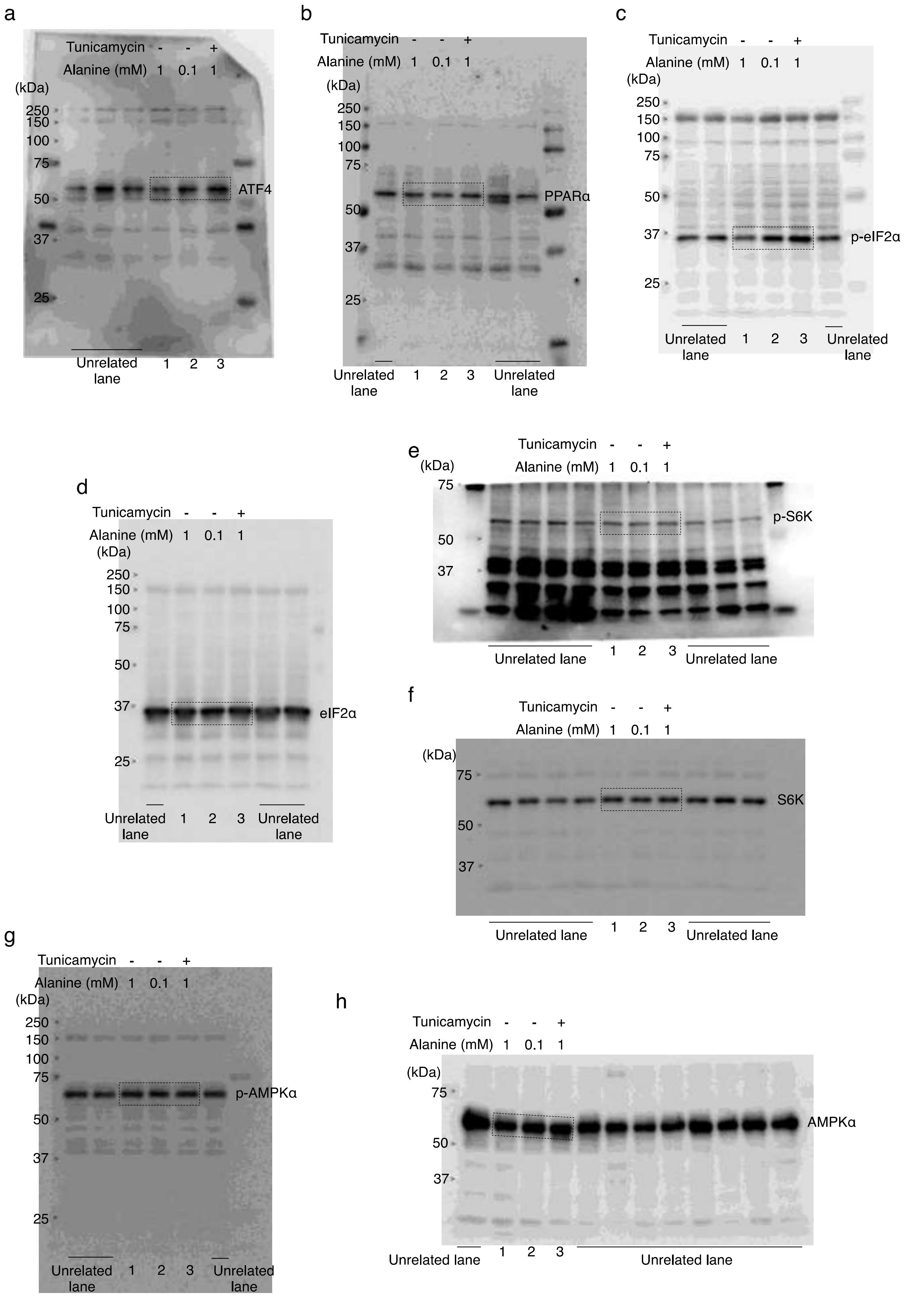

Supplementary Figure 6

Uncropped immunoblots.

(**a**-**h**) Representative immunoblots of nuclear extracts (**a**, **b**) and cytoplasmic fractions (**c**-**h**) from mouse primary hepatocytes prepared and cultured as described in the legend for **Fig. 8b**. Antibodies for blotting are indicated at the right of each panel. Positions of molecular size markers are indicated at the left of each panel. Dotted boxes indicate positions of cropped data which are presented in **Fig. 8c**.

Supplementary Table 1. PCR Primers used in this study

**Primers for genotyping**		
**Name**	**Sequence of primer (5'-3')**	
Cre1	aaatactctgagtccaaaccgggcccc	
Cre2	cagtgcgttcgaacgctagagcctgtt	
G1	ggcatgcacaattacggccttct	
G4	gtgtagcagccagcttacagga	
G8	ccttctcattccatgtcagcatgt	
**Primers for quantitative reverse transcription PCR (qRT-PCR)**		
**Gene**	**Forward primer (5'-3')**	**Reverse primer (5'-3')**
36B4	actggtctaggacccgagaag	ctcccaccttgtctccagtc
GR	tgacgtgtggaagctgtaaagt	catttcttccagcacaaaggt
KLF15	acaggcgagaagccctttt	catctgagcgggaaaacct
FoxO1	cttcaaggataagggcgaca	gacagattgtggcgaattga
FoxO3a	gctaagcaggcctcatctca	ttccgtcagtttgagggtct
FoxO4	aaggacaagggtgacagcaa	ctgtgcaaggacaggttgtg
MuRF1	cctgcagagtgaccaagga	ggcgtagagggtgtcaaact
atrogin-1	agtgaggaccggctactgtg	gatcaaacgcttgcgaatct
Bnip3	cctgtcgcagttgggttc	gaagtgcagttctacccaggag
LC3	catgagcgagttggtcaaga	ccatgctgtgctggttga
REDD1	ccagagaagagggccttga	ccatccaggtatgaggagtctt
Fkbp5	aaacgaaggagcaacggtaa	tcaaatgtccttccaccaca
HSL	ccatagtcaagaaccccttca	atctagcatggggtccagag
ATGL	tgaccatctgccttccaga	tgtaggtggcgcaagaca
SREBP1c	cgggacagcttagcctctac	ggtacgggccacaagaagta
FASN	gcagtcctgagcagctttgt	cgaggtctcggatgccta
DGAT2	ggcgctacttccgagactac	tggtcagcaggttgtgtgtc
PGC-1α	gaaagggccaaacagagaga	gtaaatcacacggcgctctt
Ucp1	ggcctctacgactcagtcca	taagccggctgagatcttgt
Cidea	aaaccatgaccgaagtagcc	aggccagttgtgatgactaagac

To be continued to the next page

Continued from the previous page

**Primers for qRT-PCR (continued)**		
**Gene**	**Forward primer (5'-3')**	**Reverse primer (5'-3')**
FGF21	cacaccgcagtccagaaag	tgacacccaggatttgaatg
ALT1	ccttcaagcagtttcaagca	gctccgtgagtttagccttg
ALT2	tatgcgttccctcggattc	ggagccattttatgggactg
cpt1a	gcagtcgactcacctttcct	atttctcaaagtcaaacagttcca
cpt1b	cccaaaacagtatcccaatcat	taagagaccccgtagccatc
cpt2	ccaaagaagcagcgatgg	tagagctcaggcagggtga
CACT	ttgtacaaagggttcaatgcag	ggcaatttcaaagccaagg
Fatp1	aaggttcttgcatcctatgctc	tggatcttgaaggtgcctgt
TAT	ggaggaggtcgcttcctatt	gccactcgtcagaatgacatc
Pck1	gatgacattgcctggatgaa	cgttttctgggttgatagcc

**Primers for chromatin immunoprecipitation (ChIP)**		
**Name**	**Forward primer (5'-3')**	**Reverse primer (5'-3')**
TIS (FGF21 +2/+88)	gacagccttagtgtcttctc	tgggtcaggttcagactgg
Coding (FGF21 +682/+761)	catgcactcccccttggc	cccttcacatatgtcaagatg
Control (FGF21 -6614/-6551)	tcagcatgcctccaaagc	tcagccttgaggaagagtagaca
ATF4RE1 (FGF21 -1060/-981	gcaggacgctgtctggtg	gcttagcattcgggccttg
ATF4RE2 (FGF21 -168/-81)	ttcagacccctgttggaaag	cacacttggcaggaacctgaat
PPRE1 (FGF21 -998/-923)	aaggcccgaatgctaagc	agcccagcaggtggaagtct
PPRE2 (FGF21 -109/-32)	cggtggaattcaggttcctg	agacaggcccgcccacg

Supplementary Table 2. Antibodies used in this study

Antibodies for immunoblotting			
**Antigen**	**Supplier**	**Catalogue number**	**Dilution**
GR	SCB	sc-1002	1:500
S6K1	SCB	sc-230	1:1000
ATF4 (CREB2)	SCB	sc-200	1:2000
GAPDH	SA	G6545	1:50000
actin	SA	A2103	1:50000
PPARα	Abc	ab2779	1:2000
4E-BP1	CST	#9452	1:1000
p-S6K1 (T389)	CST	#9205	1:1000
p-4E-BP1 (T37/46)	CST	#2855	1:1000
p-mTOR (S2448)	CST	#5536	1:1000
mTOR	CST	#2972	1:1000
p-eIF2α (S51)	CST	#3398	1:2000
eIF2α	CST	#5324	1:2000
p-AMPKα (T172)	CST	#2535	1:1000
AMPKα	CST	#2532	1:10000

Antibodies for ChIP			
**Antigen**	**Supplier**	**Catalogue number**	**Dilution**
RNA polymerase II	Cov	MMS-126R	5 µg IgG for 3.5 x 10^6 cells
ATF4 (CREB2)	SCB	sc-200	5 µg IgG for 3.5 x 10^6 cells
PPARα	Abc	ab2779	5 µg IgG for 3.5 x 10^6 cells

Antibodies for indirect immunofluorescent staining				
**Antigen**	**Supplier**	**Catalogue number**	**Clone**	**Dilution**
collagen type IV	Mil	AB756P	-	1:200
type IIb myosin heavy chain	OG	-	BF-F3	1:5
type IIa myosin heavy chain	OG	-	SC-71	1:20
type I myosin heavy chain	OG	-	BA-D5	1:60

Abbreviations used in Table 2: SCB (Santa Cruz Biotechnology, Santa Cruz, CA); SA (Sigma-Aldrich, St. Louis, MO); CST (Cell Signaling Technology, Danvers, MA); Cov (Covance, Princeton, NJ); Mil (Millipore, Billerica, MA); and OG (Our group)
